# Spectacle: fast chromatin state annotation using spectral learning

**DOI:** 10.1186/s13059-015-0598-0

**Published:** 2015-02-12

**Authors:** Jimin Song, Kevin C Chen

**Affiliations:** Department of Genetics and BioMaPS Institute, Rutgers University, 145 Bevier Road, Piscataway, NJ USA

## Abstract

Epigenomic data from ENCODE can be used to associate specific combinations of chromatin marks with regulatory elements in the human genome. Hidden Markov models and the expectation-maximization (EM) algorithm are often used to analyze epigenomic data. However, the EM algorithm can have overfitting problems in data sets where the chromatin states show high class-imbalance and it is often slow to converge. Here we use spectral learning instead of EM and find that our software Spectacle overcame these problems. Furthermore, Spectacle is able to find enhancer subtypes not found by ChromHMM but strongly enriched in GWAS SNPs. Spectacle is available at https://github.com/jiminsong/Spectacle.

**Electronic supplementary material:**

The online version of this article (doi:10.1186/s13059-015-0598-0) contains supplementary material, which is available to authorized users.

## Background

Identifying regulatory elements in the human genome is a challenging problem that is important for understanding many fundamental aspects of biology, including the molecular mechanisms of disease, development and evolution. Cell-type-specific gene regulation clearly cannot be explained by genome sequence alone because the genome is essentially identical in almost all cell types. The epigenome refers to the complete set of chromatin modifications across the entire genome, including DNA methylation marks and post-translational histone modifications, and it has received great interest in recent years for its potential to elucidate gene regulation. It has been called the ‘second dimension of the genome’ [1], and we use the term here as commonly done with no requirement for the epigenetic marks to be heritable.

Epigenetic marks are known to be correlated with fundamental biological processes such as mRNA transcription, splicing, DNA replication and DNA damage response (reviewed in [1-3]). Although it is debated whether epigenetic marks are mechanistically required for these processes, genome-wide studies have nonetheless been highly successful in using epigenetic marks to identify important genomic features that were often previously very difficult to find by other methods, including enhancers, promoters, transcribed regions, repressed regions of the genome and non-coding RNAs (e.g. [4,5]). There is also the potential to use epigenome maps to identify subclasses of functional elements, such as promoters, that are active in a cell type versus those that are poised for activation at a later time in development [6]. Importantly, functional elements identified by epigenetic marks have been shown to overlap significantly with disease-associated SNPs found by genome-wide association studies (GWASs) [7,8]. Since approximately 90% of GWAS SNPs are thought to be located in non-coding regions [9], such results give hope that one might be able to fine-map the causal disease variants of many GWASs or other disease gene mapping studies using epigenome maps.

Recently, the ENCODE project [4] produced a wealth of epigenomic data from many different human cell types using a combination of stringent biochemical assays and high-throughput sequencing technologies. In addition, the International Human Epigenome Consortium [10] also aims to produce reference maps of 1000 human epigenomes and it includes several major projects, such as BLUEPRINT and the Roadmap Epigenomics Project [11,12], which is producing epigenome maps from multiple primary human tissues. Finally, individual research labs are also producing epigenome maps for related species such as the mouse and pig [13], and for different human individuals [14]. As the number of human epigenomic data sets grows, the need for fast and robust computational methods for analyzing these data will increase.

One successful computational approach for analyzing epigenomic data is to build a unified statistical model to decipher the patterns of multiple chromatin modifications in a cell type, rather than analyzing each chromatin modification individually. Several computational methods have been developed to annotate chromatin states from epigenomic data, not only in the human genome but also in the *Drosophila*, mouse, yeast and *Arabidopsis thaliana* genomes [15-27]. Among these methods, hidden Markov models (HMMs) have been commonly used as the underlying probabilistic model of the sequence of chromatin states along the genome. Currently, a detailed understanding of the specific chromatin modifications associated with different classes of regulatory elements, such as enhancers and promoters, is lacking, so many researchers have taken the approach of performing unsupervised estimation of the HMM parameters (i.e. inferring the relevant subclasses of chromatin states directly from the data without access to existing biological examples of such subclasses). To perform unsupervised learning, the expectation-maximization (EM) algorithm has been the standard algorithm used in practice for a long time [28,29].

The EM algorithm is a maximum likelihood approach that iteratively converges to a local optimum in the likelihood. However, it suffers from several well-known issues. It is often slow to converge since the likelihood is not convex in general and EM is a first-order optimization method, and deciding when to stop the iterations is somewhat arbitrary. EM is not guaranteed to find a global optimum, so often multiple parameter initializations are needed to achieve good practical performance [30]. Finally, the maximum likelihood approach is known to be prone to overfitting [31] and to perform poorly on classification problems that suffer from class imbalance [32]. Importantly, the observation about class imbalance implies that for a data set where the majority of the genome is in the background null chromatin state without any chromatin immunoprecipitation sequencing (ChIP-seq) peaks for chromatin marks, the EM algorithm will tend to devote more parameters to modeling the large background class, at the expense of modeling other types of biologically important functional classes. All of these issues lower the biological significance of the solutions obtained from the algorithm.

To address all of these issues, here we investigated the feasibility of using spectral learning, an approach that is currently being developed in the theoretical machine learning community (e.g. [33,34]) to perform chromatin state annotation instead of the EM algorithm. Spectral learning fits in the overall framework of the method of moments or the plug-in estimator approach, which predates the maximum likelihood approach [35,36]. Broadly speaking, method of moments estimators are different by nature from maximum likelihood estimators. Instead of attempting to find the maximum likelihood solution, in the method of moments one expresses various unobservable moments of the model as functions of the parameters, sets these moments equal to the sample moments estimated from the data and solves these equations to estimate the parameters. Method of moments estimators are often simpler, faster and more efficient to compute. They also often do not suffer from local optima issues, so they do not depend on the parameter initialization.

On the other hand, maximum likelihood and method of moments estimators are complementary approaches and there are important advantages to the maximum likelihood approach. Maximum likelihood approaches are generally more sample efficient (i.e. they do not require as much data to produce the same quality of solution). Thus a common way to combine the advantages of the two methods is to use the method of moments estimator as an initializer and to run a few iterations of local optimization of the likelihood (e.g. using the EM algorithm) [37]. We hypothesized that the human genome might be large enough to provide a sufficient number of samples to compute a method of moments estimator accurately.

Thus, in this work, our main technical contribution is to develop a practical implementation of spectral learning for HMMs for the specific biological application of annotating chromatin states in the human genome. Although there are a few practical implementations of spectral learning in the natural language processing and computer vision communities, as well as a few previous implementations for specific biological problems, we believe Spectacle is one of the first practical implementations for a commonly studied biological problem. We stress that many technical issues remain to be studied in spectral learning, as described in this paper.

We found that our method, Spectacle (Spectral Learning for Annotating Chromatin Labels and Epigenomes), was much faster than a previous state-of-the-art method, ChromHMM [15], for commonly used numbers of chromatin states and epigenetic marks in a number of ENCODE cell types. We believe this speed-up will become more important as the number of new epigenomic data sets increases. In addition, we observed empirically that Spectacle was more robust to the class imbalance problem and produced fewer null chromatin states and more functional chromatin states than the EM algorithm. We believe this is a novel observation for spectral learning, which may be significant for future algorithmic work in spectral learning. Most importantly, we demonstrate the biological significance of our chromatin state annotations by showing a higher enrichment for disease-associated SNPs in the additional functional chromatin states found by Spectacle compared to ChromHMM. We analyzed selective constraints on chromatin states, which suggests that epigenome-based enhancer predictions are more informative for fine-mapping disease-associated SNPs than evolutionary conservation. Finally the faster run time of our program enabled us to compare epigenomic data sets across cell types by stacking the chromatin mark data from different cell types. This analysis suggested that most enhancer states are cell-type specific and that this set of enhancers is important for fine-mapping disease-associated SNPs. We implemented Spectacle in Java and Python by modifying the ChromHMM code, so it should be easily portable to most desktops and the source code is freely available online.

## Results

We use HMMs to represent the chromatin states as hidden states and all possible combinations of epigenetic marks as observations. We compare our software, Spectacle (see the details in the Materials and methods), which implements a spectral learning algorithm for parameter estimation, to a previous state-of-the-art method using the EM algorithm, ChromHMM.

### Spectral learning is much faster than expectation-maximization for common numbers of chromatin states and epigenetic marks

**Figure 1:**
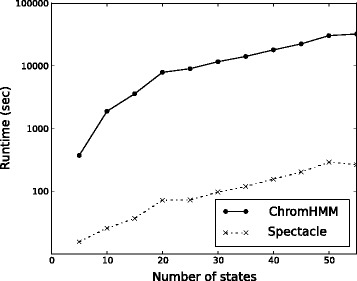
**Training time of Spectacle vs ChromHMM for the GM12878 cell line.**

In our tests, Spectacle had significantly faster training times than ChromHMM (23.9 to 124.1 times faster) for all tested numbers of chromatin states (Figure 1). Indeed, our implementation of spectral learning takes much less compute time than just two iterations of the EM algorithm – much less than required for convergence. For most biological analyses, the number of chromatin states is usually set lower than 100 and for biological interpretability it is often set to around 20 states (e.g. [22]). Thus for the number of chromatin states most relevant to biological data, Spectacle was 97 times (37 vs 3599.5 s) faster for 15 states and 110 times (71.8 vs 7915.3 s) faster for 20 states than ChromHMM. The faster training times are important in allowing individual users to annotate the large numbers of epigenomic maps being produced for different species, cell types, individuals and disease states, and we expect our method to be even more valuable in the future as the number of such data sets increases with decreasing sequencing costs.

All of these reported results used eight chromatin marks from three ENCODE Tier 1 cell types that have been used in other method development studies as well. This is also roughly the number of epigenetic marks used in other studies from individual labs. It is important to note that our method considers all possible combinations of epigenetic marks, unlike other methods that assume independence between the epigenetic marks, and therefore the theoretical space and time complexity of our method grows exponentially with the number of epigenetic marks. We consider this a biologically significant feature of our approach because given the current knowledge of all the possible chromatin marks and their interactions, we feel it is important to be open to the possibility of interactions between any set of epigenetic marks. Thus we need to consider all possible combinations of epigenetic marks, which makes the (theoretical) exponential complexity unavoidable.

On the other hand, it is also important in practice that the exponential dependence is only theoretical and in fact an analysis of 16 chromatin marks from a public human epigenomics data set shows that the actual number of combinations of epigenetic marks that appear in real human epigenomics data is much smaller and scales as only a low-order polynomial with exponent in the range 1 to 2 (Additional file 1: Figures S1, S2 and S3). We found that although there are theoretically 2^16^=65,536 possible combinations of epigenetic marks, in actual practice only 9,954 combinations (approximately 15%) of the chromatin marks appear in the genome. Only 409 combinations (approximately 0.6%) of epigenetic marks accounted for more than 99% of the genome. We downloaded narrow peak calls for 29 chromatin marks in the human embryonic stem cell H9 from the Roadmap Epigenomics Project [12]. In theory, there can be up to 2^29^ combinations of chromatin marks, but we observed that there were only 186,814 combinations appearing in the genome. After eliminating singleton combinations that are likely due to noise, only 56,750 combinations appeared at least twice in the genome. This phenomenon of data sparsity is both very common in modern statistical research and entirely consistent with our biological intuition that not all possible combinations of epigenetic marks are likely to be biologically significant for directing cellular processes. Having sparse input data allows us to use sparse linear algebra routines in our method. Indeed our computational experiments with our Python implementation using sparse singular value decomposition (SVD) methods based on power iteration show that the method is very efficient in practice on the largest data sets in the Roadmap Epigenomics Project (Additional file 1).

### Spectral learning appears to be more robust to class imbalance than expectation-maximization

**Figure 2:**
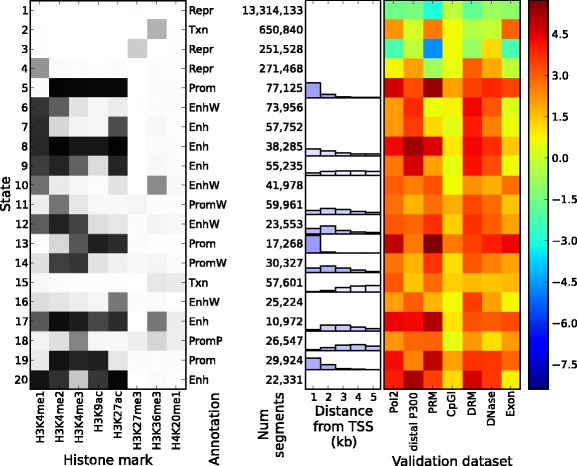
**Spectacle results for cell line GM12878 for 20 chromatin states.** **(Left)** Emission matrix and state annotation (values between 0 and 1 are colored from white to black). **(Middle)** Number of genomic segments and distance from TSS of the segments for each state. The distribution bars are shown only when at least 10% of the segments are within 5 kb from the TSS (the percentage of the segments within 5 kb from the TSS is shown by the color of the distribution bars, i.e., the more segments within 5 kb from the TSS, the bluer the distribution bars are). **(Right)** Enrichment of validated biological features (enrichment is colored red and depletion is colored blue). CpGI, CpG island; DRM, gene-distal regulatory module; Enh, strong enhancer; EnhP, poised enhancer; EnhW, weak enhancer; kb, kilobase; Num, number; Pol2, RNA polymerase II binding; PRM, promoter-proximal regulatory module; Prom, strong promoter; PromF, promoter flanking; PromP, poised promoter; PromW, weak promoter; Repr, repressed region; TSS, transcription start site; Txn, transcribed region.

**Figure 3:**
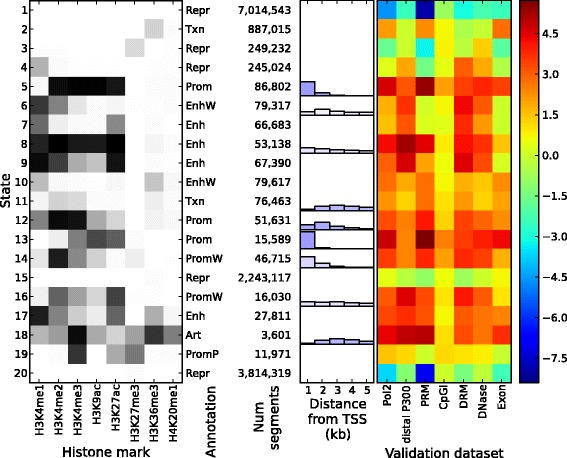
**ChromHMM results for cell line GM12878 for 20 chromatin states.** Shown are the emission matrix, state annotation and enrichment of biological features. Refer to caption of Figure 2. Art, artificial; CpGI, CpG island; DRM, gene-distal regulatory module; Enh, strong enhancer; EnhP, poised enhancer; EnhW, weak enhancer; kb, kilobase; Num, number; Pol2, RNA polymerase II binding; PRM, promoter-proximal regulatory module; Prom, strong promoter; PromF, promoter flanking; PromP, poised promoter; PromW, weak promoter; Repr, repressed region; TSS, transcription start site; Txn, transcribed region.

We examined the chromatin states inferred by Spectacle and ChromHMM for the GM12878, H1-hESC and K562 cell types, setting the number of chromatin states to 20. We observed that the EM algorithm always assigned more hidden states to modeling the background null chromatin state (Figures 2 and 3, Additional file 1: Figures S14 to S17). Figures 2 and 3 show an example of this phenomenon where states 1, 15 and 20 were all assigned the null state (i.e. all epigenetic marks with close to no signal) in the EM solution (Figure 3 left panel) whereas only state 1 was assigned to be the null state in the spectral learning solution (Figure 2 left panel). ChromHMM used the three chromatin states to cover most null segments in the genome (approximately 88%) whereas Spectacle covered about the same number of null segments (approximately 86%) with only one chromatin state.

We believe that the difference between the two approaches is due to their robustness for data with a high class imbalance. We found that using the Poisson binarization method [7] for the ChIP-seq data, the fraction of the genome in the null state was approximately 90% in all ten ENCODE cell types we examined (Table 1). It is well known that for data with a class imbalance, the maximum likelihood criterion will tend to devote more parameters to modeling the large background class (e.g. [32]). This in turn causes lower quality modeling of other biologically more interesting states, since there are fewer parameters devoted to them. For instance, state 20 is unique to the Spectacle solution and does not appear in the ChromHMM solution, yet has the hallmarks of an active enhancer (Figure 2, left panel). Similarly, for another cell type K562, Spectacle found a strong enhancer state that does not appear in the ChromHMM solution (state 20 in Additional file 1: Figure S16, left panel). We have not yet analyzed the theoretical robustness of spectral learning to class imbalance and leave it as a theoretical question for future work but speculate that it may come from the orthogonality condition in the SVD computation; the first singular vector accounts for the null state and the other vectors that account for other chromatin states are constrained to be orthogonal to it. Instead here we performed extensive empirical testing of spectral learning and EM/maximum likelihood on a more balanced epigenomics data set (Additional file 1: Figures S4 to S13, Tables S1, S2 and S3). This data set was more balanced in the sense that the fraction of the genome in the null state was approximately 52%(Table 1). For this data set, we found that maximum likelihood was indeed a good criterion that corresponds to better performance for predicting biological features, and we show that spectral learning was useful as an initializer to a local optimization method for the likelihood, a common use of method of moments estimators in statistics.

**Table 1:** **Fraction of null segments for ten ENCODE cell types**

**Binarization**	**Cell type**			
	GM12878	H1-hESC	HeLa-S3*	HepG2*
Scripture [38]	0.5	0.59	0.47	0.52
Poisson binarization [7]	0.92	0.92	0.89	0.89
	HMEC	HSMM	HUVEC	K562
Scripture [38]	0.48	0.49	0.55	0.56
Poisson binarization [7]	0.90	0.89	0.89	0.91
	NHEK	NHLF		
Scripture [38]	0.57	0.51		
Poisson binarization [7]	0.91	0.91		

Cell types with * have only seven available epigenetic marks for the Poisson binarization data set. All other cell types have eight epigenetic marks for both binarization data sets.

We note that simple approaches to addressing class imbalance, such as subsampling from the dominant class, which are used in simple classification problems (e.g. using naive Bayes classifiers), are not directly applicable in the HMM context because the length distribution of the chromatin states is important for the model and cannot simply be changed by sampling. Finally, we note that for the Poisson binarization data, when we used the spectral learning parameters to initialize the EM algorithm and ran it to convergence, in all cases the parameters converged to those found by ChromHMM. This indicates that the difference between the chromatin state annotations of ChromHMM and Spectacle are not due to the initialization method used in ChromHMM but rather a difference between the spectral learning and maximum likelihood approaches.

### Comparison of chromatin state annotations

Next we studied the biological significance of the chromatin state annotations produced by the two methods. To do so, we did an in-depth study for three ENCODE Tier 1 cell types: GM12878, H1-hESC and K562. We ran both Spectacle and ChromHMM using eight epigenetic marks for 20 states and annotated the predicted chromatin states from the eight epigenetic marks as promoters (Prom), enhancers (Enh), transcribed regions (Txn) and repressed regions (Repr). We then validated the predicted chromatin states using external biological data sets.

#### Promoters

In the literature, promoters are characterized by high H3K4me3/H3K4me2 and low H3K4me1, while H3K9ac is associated with promoter activity (reviewed in [39,40]). Promoter states are categorized into three states depending on enrichment of H3K4me3/H3K4me2 and H3K9ac, or H3K36me (signal for flanking regions): Prom (strong promoter), PromW (weak promoter) and PromF (promoter flanking). For the GM12878, H1-hESC and K562 ENCODE Tier 1 cell types, we found 5, 3 and 5 Spectacle promoter states and 5, 3 and 4 ChromHMM promoter states, respectively. These segments were associated with active transcription start sites (TSSs) (Table 2). Also, we found that these were highly enriched with relevant biological features including RNA polymerase II binding (Pol2), promoter-proximal regulatory modules (PRMs), CpG islands (CpGIs), DNase 1 hypersensitive sites (DNase) and exonic regions (exons), and tend to be close to active TSSs (Figures 2 and 3, Additional file 1: Figures S14 to S17). These results confirmed the accuracy of our promoter annotations with both Spectacle and ChromHMM.

**Table 2:** **Precision and recall for predicting active transcription start sites**

**Cell line**	**Data set**	**Spectacle**	**ChromHMM**
GM12878	TSS	0.045, 0.937	0.044, 0.930
H1-hESC	TSS	0.090, 0.866	0.082, 0.895
K562	TSS	0.059, 0.917	0.065, 0.922

TSS, transcription start site.

Next we examined the differences between the ChromHMM and Spectacle annotations. We found that Spectacle predicted an interesting pattern of epigenetic marks, which did not appear in the ChromHMM solution. Specifically, Spectacle state 19 for GM12878 (Figure 2) had a pattern of high H3K4me3, high H3K9ac and low H3K27ac, and was highly enriched with active TSSs (log2 of fold enrichment: 5.7). ChromHMM state 12 (Figure 3) had a similar pattern but slightly lower H3K9ac, and was less enriched with active TSSs (log2 of fold enrichment: 2.6).

There was 1, 1 and 0 poised promoter state (PromP; defined by high enrichment of H3K27me3) in Spectacle, and 1, 1 and 0 poised promoter state in ChromHMM for GM12878, H1-hESC and K562, respectively. It has been shown that these bivalent states are mostly found in embryonic stem cells [6] but are found in other cell types as well and are associated with genes involved in development in other cell types [3,41]. In fact, these states are enriched with gene ontology (GO) terms related to development and differentiation according to the GREAT software [42] (see Materials and methods for detailed parameter settings). For GM12878, Spectacle chromatin state 18 was associated with several development and differentiation-related GO terms such as ‘digestive tract mesoderm development’ (*q*<4×10^−7^), whereas ChromHMM chromatin state 19 was not associated with any such GO terms. For H1-hESC, both Spectacle and ChromHMM poised promoter states were associated with development and differentiation-related GO terms, e.g. Spectacle state 18: ‘myeloid progenitor cell differentiation’ (*q*<2×10^−4^) and ChromHMM state 13: ‘central nervous system neuron differentiation’ (*q*<5×10^−50^).

Taken together, both methods effectively identified active and inactive promoter states. However, Spectacle found certain biologically relevant chromatin states not found by ChromHMM and we believe this is because maximum likelihood methods tend to use more parameters to model the null chromatin state, which makes up the vast majority of the genome.

#### Enhancers

Enhancers are characterized in the literature by high H3K4me1/H3K4me2 and low H3K4me3, while H3K27ac is associated with enhancer activity (reviewed in [39,40]). Enhancer states are categorized into three states depending on enrichment of H3K4me1/H3K4me2 and H3K27ac: Enh (strong enhancer), EnhW (weak enhancer) and EnhP (poised enhancer; with high H3K27me3). There were 9, 9 and 10 enhancer states in Spectacle and 6, 7 and 8 enhancer states in ChromHMM for GM12878, H1-hESC and K562, respectively. These enhancers were associated with distal P300 peaks, gene-distal regulatory modules (DRMs) and VISTA enhancers (Materials and methods) as well (Table 3). They were also highly enriched with Pol2, distal P300, DRM and DNase signals (Figures 2 and 3, Additional file 1: Figures S14 to S17).

**Table 3:** **Precision and recall for predicting distal P300, gene-distal regulatory modules and VISTA enhancers**

**Cell type**	**Data set**	**Spectacle**	**ChromHMM**
GM12878	Distal P300	0.129, 0.421	0.116, 0.407
	DRM [43]	0.190, 0.375	0.174, 0.369
	VISTA	0.001, 0.048	0.001, 0.053
H1-hESC	Distal P300	0.102, 0.477	0.144, 0.342
	DRM [43]	0.115, 0.468	0.160, 0.332
	VISTA	0.004, 0.273	0.005, 0.149
K562	Distal P300	0.060, 0.204	0.057, 0.196
	DRM [43]	0.039, 0.178	0.039, 0.181
	VISTA	0.002, 0.133	0.002, 0.129

Note that VISTA enhancers are not specific to these cell types.

DRM, gene-distal regulatory module.

Importantly, we found that Spectacle could subclassify active enhancers better than ChromHMM. For instance, for GM12878, most segments in ChromHMM state 9 were labeled with either state 9 or state 20 in Spectacle according to H3K9ac activity.

For H1-hESC, some enhancers were poised enhancers with high H3K27me3. There were five Spectacle and one ChromHMM poised enhancer states. Poised enhancer states were also highly enriched with Pol2, distal P300, DRM and DNase signals, similar to active enhancer states (Additional file 1: Figures S14 and S15) [44]. All poised enhancer states found by the two methods were associated with several development and differentiation related GO terms, e.g. Spectacle state 12: ‘sensory organ development’ (*q*<1×10^−66^), Spectacle state 13: ‘enteric nervous system development’ (*q*<4×10^−6^), Spectacle state 16: ‘positive regulation of neuron differentiation’ (*q*<6×10^−3^), Spectacle state 19: ‘cell differentiation in spinal cord’ (*q*<3×10^−19^), Spectacle state 20: ‘tissue development’ (*q*<2×10^−23^) and ChromHMM state 12: ‘skeletal system development’ (*q*<1×10^−70^). We found that Spectacle subclassified enhancers depending on their distance from the TSS. Spectacle states 12, 13 and 19 were closer to the TSS but Spectacle states 16 and 20 were farther from the TSS (Additional file 1: Figures S14 and S15).

Taken together, Spectacle found more biologically relevant subclasses of enhancers than ChromHMM. We will discuss the biological significance of these enhancer subtypes further in the next section with regards to GWAS SNPs.

#### Transcribed regions

We define transcribed regions or gene bodies based on enrichment with H3K36me3. There were 2, 2 and 1 transcribed region states (Txn) in Spectacle and 2, 1 and 2 transcribed region states in ChromHMM for GM12878, H1-hESC and K562, respectively. We found that all these states were highly enriched with annotated exons.

#### Repressive and quiescent regions

The majority of the genome is considered to be in a repressed state (Repr) since there is very low enrichment of histone marks. There are a few segments (usually <0.03% of the genome) that have high enrichment of almost all epigenetic marks. These segments are considered artificial (Art) and are also treated as repressed regions. There were 3, 5 and 4 repressed states in Spectacle and 6, 8 and 6 null (Repr and Art) states in ChromHMM for GM12878, H1-hESC and K562, respectively. Thus ChromHMM devoted more of the parameters to modeling the repressed state than Spectacle, consistent with our observations about class imbalance.

Taken together, our chromatin annotation shows that ChromHMM devoted more states to modeling the background repressed chromatin state whereas Spectacle devoted more states to modeling other states, which we have argued are biologically significant.

### Genome-wide association study SNPs are enriched in strong enhancer states

Next we investigated if any of the chromatin enhancer states discovered by Spectacle were significantly enriched in disease-associated SNPs discovered in GWASs. To do so, we downloaded SNPs from the NHGRI GWAS catalogue [9]. Since approximately 90% of GWAS SNPs are found in non-coding regions of the genome, epigenomic data has been suggested as a way to give functional interpretation for non-coding GWAS SNPs [45-47]. Chromatin state annotation tools can be used to annotate strong enhancers in the genome and predict possible functions for the disease-causing SNPs as disrupting regulation of specific genes [7,22]. After excluding SNPs in unmapped chromosomes or chrY and phenotypes (diseases or traits) with less than ten SNPs, our final data set consisted of 10,604 non-coding SNPs associated with 360 phenotypes.

Overall, we found that the non-coding SNPs were highly enriched in the strong enhancer states predicted by either Spectacle or ChromHMM (*χ*^2^ test: *P*=0 for all three cell lines). The enrichment in Spectacle strong enhancer states was similar to but slightly higher than in ChromHMM strong enhancer states (log2 of fold enrichment for Spectacle vs ChromHMM: GM12878: 1.35 vs 1.32; H1-hESC: 1.56 vs 1.41; K562: 1.17 vs 1.11; all *P*>0.05).

Next we computed the fold enrichment of the SNPs for each phenotype in each chromatin state separately, since the common use of chromatin state annotations is to consider one GWAS data set at a time. In total, we found 102 phenotypes whose SNPs were most highly enriched in one of the strong enhancer states predicted by either Spectacle or ChromHMM for GM12878. Of these states, Spectacle chromatin state 20, which did not appear in ChromHMM, had the largest number of phenotypes and largest total number of GWAS SNPs, which were most highly enriched in it (Table 4). This result suggests that spectral learning may be able to produce more biologically meaningful chromatin state annotations for mapping GWAS SNPs. Methodologically, this is presumably because the enhancer subclasses account for only a small fraction of the genome, and therefore are not influential in the maximum likelihood solution, but distinctive in terms of their pattern of chromatin marks, and therefore are found by the spectral learning algorithm. More importantly, it suggests that we may have found a biologically meaningful enhancer subtype using our spectral learning approach.

**Table 4:** **Most highly enriched phenotypes for GM12878**

**Method**	**State**	**Number of**	**Number of**	**Number of**
		**phenotypes**	**associated SNPs**	**autoimmune**
				**phenotypes**
Spectacle	7	16	530	0
Spectacle	8	20	732	6
Spectacle	9	12	290	0
Spectacle	17	6	276	0
Spectacle	20	29	1284	10
ChromHMM	7	5	142	0
ChromHMM	8	2	35	0
ChromHMM	9	2	28	0
ChromHMM	17	10	370	0

Number of phenotypes whose SNPs were most highly enriched in a strong enhancer state, number of all non-coding SNPs associated with the phenotypes, and number of autoimmune disease-related phenotypes among the most highly enriched phenotypes in GM12878.

**Figure 4:**
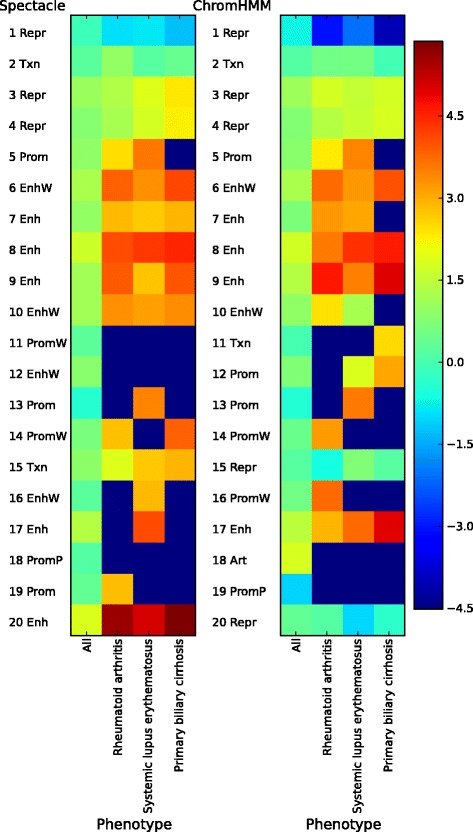
**Chromatin states enriched with GWAS SNPs for autoimmune diseases for GM12878.** Art, artificial; Enh, strong enhancer; EnhW, weak enhancer; GWAS, genome-wide association study; Prom, strong promoter; PromP, poised promoter; PromW, weak promoter; Repr, repressed region; SNP, single nucleotide polymorphism; Txn, transcribed region.

Further, we looked into phenotypes related to autoimmune diseases. GM12878 is infected with Epstein–Barr virus and it has been shown that infection with the virus is related to the risk of certain autoimmune diseases [48,49]. We found that 16 out of the 21 autoimmune disease-related phenotypes were most highly enriched in strong enhancers predicted by either Spectacle or ChromHMM, and in particular all of the 16 phenotypes in strong enhancer states in Spectacle but not in ChromHMM. Notably, we found that chromatin state 20 in Spectacle (Figure 2) had the highest enrichment of GWAS SNPs for ten out of the sixteen autoimmune diseases including all three phenotypes that were shown to be enriched in strong enhancer states in a previous work [7] (Figure 4 for the three phenotypes studied previously, Additional file 1: Figure S18 for all 16 phenotypes). This suggests that this chromatin state, which was found only by Spectacle, might be particularly interesting for mapping causing variants of disease phenotypes in this cell type.

The genomic segments labeled with Spectacle chromatin state 20 were mostly found in ChromHMM chromatin state 9. Most genomic segments annotated as enhancer state 9 in ChromHMM were divided into two enhancer states, 9 and 20, in Spectacle according to their enrichment of H3K9ac (Figure 2). This result is biologically significant as it shows that an unbiased analysis of the epigenomic data for this cell type found this particular distinction of chromatin states to be statistically important for explaining the patterns of chromatin marks and it is consistent with previous reports that H3K9ac separates stronger enhancers from weaker enhancers [50]. It would be expected that if a chromatin state were randomly split in two then one of the states might have higher enrichment by chance, but we observed that Spectacle state 20 had much more enriched phenotypes and SNPs than Spectacle state 9 (e.g. 1284 vs 290 SNPs). Our results were robust whether or not we controlled for the well-studied major histocompatibility region (e.g. following [51]), which can skew the statistics we computed.

We found similar results on GWAS SNP enrichment analysis for the K562 cell line to those for the GM12878 cell line. There were 98 phenotypes whose SNPs were most highly enriched in one of the strong enhancer states predicted by either Spectacle or ChromHMM for K562, an erythroleukemic cell line. Of these states, chromatin state 20 from Spectacle, which was not found by ChromHMM, had the largest number of phenotypes and largest total number of GWAS SNPs, which were most highly enriched in it over all strong enhancer states found by either method (Additional file 1: Table S5). Like previous analysis, we took the union of all the SNPs for all the phenotypes to account for correlations between the phenotypes (for example, two phenotypes could be similar).

Most segments labeled with ChromHMM enhancer state 7 were divided into two Spectacle enhancer states, 7 and 20, according to the enrichment of H3K36me3 (Additional file 1: Figure S16). Notably, five out of the six phenotypes related to erythrocytes were most highly enriched in Spectacle state 20 (Additional file 1: Figure S19). Specifically the enrichment in Spectacle state 20 was higher than the closest strong enhancer state annotated by ChromHMM for the five erythrocyte-related phenotypes related to the cell type we are studying. Additional file 1: Table S5 shows that there are many more phenotypes and SNPs more highly enriched in Spectacle state 20 compared to Spectacle state 7 (e.g. 1,614 SNPs in Spectacle state 20 vs 436 SNPs in Spectacle state 7). This suggests that Spectacle state 20 may be better for identifying potential causal variants in this cell type than other annotated strong enhancers.

We repeated the GWAS analysis for the H1-hESC cell type but there was no difference between the strong enhancer state annotations of Spectacle and ChromHMM, so we did not investigate the enrichment of GWAS SNPs further (Additional file 1: Table S4).

### Analysis of selective constraint at the population genetic and cross-species levels

Next we quantified the levels of selective constraint acting on the Spectacle chromatin states at the population genetic and cross-species levels. For each chromatin state we computed the fraction of SNPs with minor allele frequency (MAF) less than 0.1 at the population level, and the mean of phastCons scores at the cross-species level (see more details in Materials and methods). We used these data to analyze the levels of conservation of the different chromatin states (Additional file 1: Tables S7 and S8). In general, we found a reasonable concordance between the population genetic and cross-species measures. We found that promoter and transcribed states tended to be under the strongest negative selection while repressive and quiescent (null) states tended to be under the weakest negative selection, consistent with our biological intuition. Enhancer states tended to be under intermediate levels of negative selection, at least at the SNP level. Among enhancer states, those with epigenomic marks associated with either promoters (H3K4me3) or transcribed regions (H3K36me3) tended to be under stronger negative selection than the other enhancers. Enhancer state 20 in Spectacle, which showed high enrichment for GWAS SNPs for GM12878, was under less negative selection than enhancer state 9 (Mann–Whitney U test: Spectacle state 20 vs Spectacle state 9: *P*<4.1×10^−10^). This is a biologically plausible result since disease-causing alleles might be under intermediate levels of selective constraint compared to neutral (i.e. non-functional) alleles and highly constrained (i.e. lethal) alleles. In addition, we noticed that one transcribed state (Spectacle state 15 of GM12878) was one of the most selectively constrained states at the population genetic level but one of the least selectively constrained at the cross-species level. This pattern is consistent with the action of recent species-specific selection. Since this chromatin state does not strongly correspond to cell-type-specific protein-coding exons or long non-coding RNAs (log2 fold enrichment: 1.7 for exons and 1.5 for long non-coding RNAs), it presumably corresponds to a class of lowly expressed and/or poorly understood non-coding transcripts. The action of recent selection on this class of transcripts is again consistent with our biological intuition.

Overall, our results also suggest more general principles about the informativeness of different types of functional annotation for fine-mapping GWAS SNPs. We have shown that enhancer states annotated from global epigenomics data are highly enriched for GWAS SNPs despite not being under strong negative selection. We compared Spectacle chromatin state 20 of GM12878 with the most conserved phastCons intergenic regions in terms of GWAS SNP enrichment (we selected the same number of conserved genomic segments as in chromatin state 20). The log2 fold enrichment in Spectacle state 20 for GM12878 vs most conserved regions for all SNPs was 1.87 vs 0.20. Spectacle state 20 of GM12878 had 40 phenotypes with positive log2 fold enrichment and 14 out of the 40 phenotypes were autoimmune-related phenotypes, whereas the most conserved regions had 16 phenotypes with positive log2 fold enrichment and none of them were autoimmune-related phenotypes. Similarly, we compared Spectacle chromatin state 20 of K562 with the most conserved phastCons regions. The log2 fold enrichment in Spectacle state 20 of K562 vs most conserved regions for all SNPs was 1.59 vs −0.32. Spectacle state 20 of K562 had 34 phenotypes with positive log2 fold enrichment and 6 out of the 34 phenotypes were erythrocyte-related phenotypes, whereas the most conserved regions had nine phenotypes with positive log2 fold enrichment and none of them were erythrocyte-related phenotypes. These results suggest that epigenomics-based enhancer predictions may be more informative for fine-mapping GWAS SNPs than looking for evolutionarily conserved regions. A similar result was previously found for microRNA binding sites where predicted conserved microRNA binding sites were shown to be more informative of selective constraint than searching for conserved 3 ^′^ UTR regions [52].

### Cell-type specificity of chromatin states

Finally, we analyzed the cell-type specificity of chromatin states by performing a combined analysis of epigenomics data from multiple cell types. In a previous approach, epigenomics data from nine human cell types were concatenated and the same HMM parameters were learned for the nine human cell types by running ChromHMM for the virtual long genome [7]. However, there are some disadvantages to characterizing cell-type specificity of chromatin states in this way. First, this approach produces a single set of parameters for all cell types, which might not characterize chromatin states existing in a single cell type (e.g. a bivalent state in an embryonic stem cell [6]). Second, it is non-trivial to interpret the chromatin states of the genomic segments from multiple cell types from the concatenated data sets in a post-processing step since the genome segmentations of the different cell types need to be aligned and compared.

The TreeHMM paper [21] suggested a more complicated model that utilized the lineage information between multiple cell types instead of naively concatenating cell types. However, since the model is more complicated, a variational approximation was used for parameter learning, which is technically an unbounded approximation of the EM algorithm and thus may not always give accurate biological results. The model also requires that the relationships between cell types are well described by a tree and more importantly that the tree is known beforehand.

We took a different approach of stacking epigenome data sets from multiple cell types to perform a joint analysis of multiple cell types, similar to [24]. By doing so, we can learn a single set of chromatin states with a uniform genome segmentation for the different cell types. We stacked three cell types with eight histone marks each for GM12878 and H1-hESC and seven histone marks for HepG2 (the H3K4me1 mark for HepG2 was not available). There were 79,839 combinations of the 23 histone marks in the genome. If each cell type had seven chromatin states, there would be a total of 7^3^=343 possible combined chromatin states for the three cell types. To improve biological interpretability and running time, we ran Spectacle with 50 combined chromatin states (Additional file 1: Figure S22), which is still a large number that would be slow to run using the EM algorithm. Consistent with previous results [7,21], we found that most enhancers were cell-type specific while promoter states could be cell-type specific or constitutive across cell types. We confirmed the cell-type-specific enhancer states had a distal P300 signal for only the corresponding cell type and lacked the TSS signal. Promoter states that were either constitutive or cell-type specific had a corresponding enrichment of the TSS signal.

Importantly, we found that higher enrichment of non-coding GWAS SNPs in strong enhancer states was specific to the cell-type-specific enhancer states of the GM12878 cell line (Additional file 1: Figure S22, Table S9). Among 132 phenotypes whose SNPs were most highly enriched in one of the 13 enhancer states, 14 phenotypes were for autoimmune diseases and all of them were most highly enriched in one of the enhancer states that are specific to GM12878.

We note that running the EM algorithm (e.g. with ChromHMM) with such a large number of hidden chromatin states is computationally inefficient. It took approximately 1.6 hr for Spectacle and approximately 8.5 hr for ChromHMM. This demonstrates that our spectral learning approach facilitates the analysis of epigenome data from multiple cell types.

## Discussion

We have developed a practical, robust implementation of a published spectral learning algorithm for HMMs, which we have specially tuned to specific features of epigenomic data. We have implemented our method in a software tool called Spectacle (Spectral Learning for Annotating Chromatin Labels and Epigenomes), which we have tested extensively on human epigenomic data sets though it should be useful for many model organisms as well. We made a number of technical modifications to a previously published algorithm [33], which improved its accuracy and numerical stability on epigenomics data sets. Using the commonly used Poisson binarization of the ENCODE epigenetic mark data, we showed that Spectacle is much faster than ChromHMM for commonly used numbers of chromatin states and epigenetic marks. Furthermore, the overall statistical approach appears to be more robust to class imbalance than the usual maximum likelihood approach, which is an observation we believe to be novel for spectral learning. We support our empirical observations on class imbalance by testing our method extensively on a set of broad peak epigenetic mark data (Additional file 1: Figures S4 to S13, Tables S1, S2 and S3). These data are not commonly used in biological applications but they are useful for demonstrating the utility of our program on a data set that exhibits more class balance. For this data set, we showed that the likelihood appears to be a good optimization criterion for retrieving biologically relevant features. In this case, the spectral learning approach and local likelihood optimization methods, such as EM, are complementary. Spectral learning can be used to initialize the EM algorithm, which is a common use of method of moments estimators in statistics. In this context, we show that the spectral learning initializer usually outperforms a previously published initialization heuristic in terms of finding higher likelihoods, faster running time and higher accuracy for several independent biological data sets. This observation may be useful for chromatin state annotation in model organisms that have proportionally more coding DNA and non-coding functional regions than humans and therefore might have epigenome data sets that are more class balanced.

Overall, our software implementation is freely available online and is lightweight and easy to use on a regular desktop without the need for specialized computer hardware. Our code modifies the ChromHMM code, which has been used by several experimental groups, so we believe it will be user-friendly and accessible. Importantly, we show that although the chromatin state annotations produced by Spectacle are similar overall to those produced by ChromHMM for two out of three ENCODE Tier 1 cell types, Spectacle found enhancer subtypes that were significantly more enriched in GWAS SNPs for relevant diseases than the enhancer states found by ChromHMM. Furthermore, the fast running time of Spectacle for high numbers of chromatin states facilitated the analysis of combined epigenome data sets from multiple cell types and we found that GWAS SNPs were enriched in these cell-type-specific enhancer states. Such a refinement of the chromatin state annotations will be important for downstream biological applications as the field moves towards fine-mapping the causal variants of complex human diseases. Our population genetic and cross-species analyses of selective constraint on the Spectacle chromatin states not only revealed interesting evolutionary patterns but also suggested that enhancer predictions from epigenomics data may be more informative for fine-mapping GWAS SNPs than evolutionary conservation. We note that GWAS SNPs tend to be common in the population so it is not surprising that they are not highly enriched in evolutionarily conserved regions. For rare disease variants, the results could be different.

The refinements to the spectral learning algorithm we have described can be applied to multiple problems in computational biology and should be of broader interest. To our knowledge we have used spectral learning for one of the first times to learn HMM parameters explicitly for a problem in computational biology. A recent work used spectral learning for poly(A) motif prediction [53]. The authors did not try to recover the HMM parameters explicitly but instead learned them up to an unknown invertible linear transformation and used the transformed parameters as features for classification by a support vector machine. Another recent paper [54] applied a spectral learning algorithm for contrastive learning to a problem involving epigenome maps. This is a more restricted version of the problem we study here in which one is specifically contrasting two data sets instead of annotating a single data set. HMMs and the EM algorithm are used in many other problems in computational biology for problems as diverse as gene finding, modeling linkage disequilibrium, predicting enhancers or microRNA binding sites, and detecting copy number variation from array CGH data. Thus, it is very possible that the methods described here may be useful in those settings as well.

We provide an implementation of our method. It uses Python sparse matrix libraries to allow users to analyze a large number of chromatin marks, for example in the Roadmap Epigenomics Project. It is also possible to reduce the dimension of the observation space in the HMM using principal component analysis in a similar way to [27]. For further technical improvement, one might utilize recent developments in approximate, randomized linear algebra (e.g. using random linear projections and QR decompositions for fast SVD computations) and we leave a full exploration for future work. In addition, we stress that our overall modeling approach is fundamentally different from previous methods such as ChromHMM, which assumed independence between chromatin marks. Instead we consider all possible combinations of epigenetic marks and discover arbitrary interactions between chromatin marks for which there is statistical support. Since researchers are only at an early stage of exploring the full biological complexity of the epigenetic code, we believe this is a useful and important aspect of our approach.

## Conclusions

Here we have presented a new software tool, Spectacle, for annotating chromatin states in the genome from epigenome maps, such as those produced by ENCODE. We have developed a practical implementation of a spectral learning approach for HMMs that was previously mainly discussed in the theoretical literature and should be of broad interest for other computational biology problems. We have also demonstrated that the approach is faster and more robust to class imbalance than the more commonly used EM approach. In particular, for two out of three ENCODE Tier 1 cell types, we show that the highest enrichment of cell-type-related GWAS SNPs is in an enhancer state only found in Spectacle and not in ChromHMM. Furthermore, for both of these cell types, the enrichment of GWAS SNPs in these enhancer subtypes inferred from epigenome maps was much higher than in evolutionary conserved intergenic regions. This result may suggest a more general principle for future fine-mapping of GWAS SNPs. We also found that most enhancers appear to be cell-type specific based on our analysis of combined epigenomics data from multiple cell types and we found that GWAS SNPs were highly enriched in these cell-type-specific enhancer states.

In the future, we believe that having a faster and more robust chromatin state annotation tool should be useful for annotating multiple epigenomic maps. Previous works found associations between disease-causing variants and epigenomic marks [8,14,55,56], suggesting that a better understanding of the epigenome might help interpret the variants underlying human disease. For example, [14] used ChromHMM to infer variation in chromatin states across individuals, so we expect Spectacle to be useful for other similar types of data sets in the future. Indeed there are currently several major epigenomics projects (e.g., BLUEPRINT and the Roadmap Epigenomics Project) producing chromatin mark data for many cell types and human populations [11,12]. Given the rapid decrease in the cost of sequencing, we also expect that many more epigenomic maps will be produced for different cell types, human populations [14], species [13], environmental conditions and developmental contexts [7,57]. Thus we expect that the need for fast and robust tools for processing this type of data will continue to grow in the future.

## Materials and methods

### Related work

To identify functional elements from epigenomic data, there are two broad classes of approaches: supervised and unsupervised learning. Supervised learning associates patterns of epigenetic modifications with known classes of functional elements, such as enhancers, promoters and non-coding RNAs. It generally has good performance for known classes of functional elements but it requires the availability of independently validated examples and it cannot discover new classes of chromatin states [5,16,25,43,58]. In contrast, unsupervised learning discovers patterns of epigenetic modifications for each chromatin state directly from the data [15,18,59-62]. Here we take an unsupervised approach as we believe that the current state of the field is such that more research is needed to understand better the variety of possible chromatin states and the specific epigenetic marks underlying them. Indeed the results presented in this paper suggest that additional enhancer subtypes remain to be discovered.

One method, ChromHMM [15,62], uses a HMM to model epigenomic data where each chromosome is segmented into non-overlapping regions of 200 bp and each segment has a binary value representing the presence or absence of each epigenetic mark. Each segment is assumed to be in a hidden chromatin state, which defines a distribution over combinations of epigenetic marks. To learn the HMM parameters, ChromHMM uses the EM algorithm [28], also called the Baum–Welch algorithm in the context of HMMs. To run the EM algorithm, instead of initializing the parameters randomly, ChromHMM provides a heuristic initialization method called the information method [62]. It then runs the EM algorithm to convergence, that is, until the difference between the likelihood of the current iteration and that of the previous iteration is less than 0.001. Regardless of convergence, the maximum number of iterations was set to 200. We ran ChromHMM for training using four multiprocessors in parallel on a desktop.

Another method, Segway [18], uses a generalization of HMMs called dynamic Bayesian networks to model chromatin states. For example, Segway models the length distribution of each chromatin state by adding a hidden state called a *countdown* variable. Segway has high spatial resolution since it uses a segment size of 1 bp [22]. However, it is much slower than ChromHMM because of the high resolution and the number of parameters in the model. Nevertheless, the performance of Segway on biological data sets is similar to ChromHMM [22]. Since the state space of Segway is much larger than ChromHMM, it might be harder to find the global optimum of the likelihood. Currently, Segway cannot be run on a desktop but needs to be run on a compute cluster.

The self-organizing map (SOM) model [24] gives a finer resolution analysis of the patterns of chromatin marks. It takes a genome segmentation as input (e.g. using stacked chromatin marks from multiple cell types and running ChromHMM), discovers at least 1,000 distinct chromatin states, and visualizes the chromatin states in a two-dimensional map. Similar chromatin states are located close to each other in the map and so the SOM method is able to find interesting clusters of chromatin states. The SOM model does not need to fix the number of chromatin states ahead of time but allows a larger number of chromatin states. This fine-resolution analysis was able to find clusters enriched in specific GO terms. It is also not obvious how to focus on particular chromatin states in the map since there are so many chromatin states. Instead the user is encouraged to use the map interactively. In general, the SOM model is complementary to the Spectacle software, and Spectacle can be used instead of ChromHMM in the initial genomic segmentation.

In addition, there are a few other methods that are more focused on different aspects of analyzing epigenomic data, such as enabling joint analysis of multiple data sets (jMOSAiCS [26]) and incorporating lineage information between cell types (TreeHMM [21]). As described above, many methods use HMMs to model the chromatin states and these methods differ mainly in the way they model the epigenetic mark data. For instance, some methods discretize the data while others fit Gaussian distributions to the data. Here we did not explore different approaches to modeling the underlying ChIP-seq mark data but rather focus on exploring the properties of spectral learning in the context of chromatin state annotation. We present our results comparing Spectacle with ChromHMM in the main text and our results comparing with other methods in Additional file 1.

### Hidden Markov models and spectral learning

We discuss spectral learning from the viewpoint of theoretical machine learning and the few attempts to apply spectral learning in other applied fields in Additional file 1. Here we describe our practical implementation of spectral learning for the chromatin state annotation problem.

#### Description of the hidden Markov model

We use HMMs to represent the chromatin states as hidden states and all possible combinations of (binarized) epigenetic marks as observations. The whole genome is divided into segments of size 200 bp following [15,62]. We define the HMM in matrix form as follows. Note the matrix and vector indices in this paper. For a matrix *M*, *M*[ *i*,*j*] denotes an element in the *i*th row and the *j*th column. For a vector *v*, *v*[ *i*] denotes the *i*th element.

Let *K* be the number of hidden chromatin states and *N* be the number of possible combinations of epigenetic marks (i.e., *N*=2^*M*^ where *M* is the number of epigenetic marks). Let *A* be the state transition matrix where *A*[ *i*,*j*] is the probability of transition from state *j* to state *i* for 1≤*i*,*j*≤*K*. Let *O* be the emission matrix where *O*[ *i*,*j*] is the probability of observing the *i*th combination of the epigenetic marks in state *j* where 1≤*i*≤*N* and 1≤*j*≤*K*. Let *π* be the initial state distribution vector where *π*[ *i*] is the probability of state *i* in the first segment of each chromosome when 1≤*i*≤*K*. To simplify the description of the method, the whole genome is considered to be one chromosome by concatenating all chromosomes. Let *T* be the number of segments in the genome. Let *x*_*t*_ be the observation at the *t*th segment and let $x_{t_{1}:t_{2}}$ represent $x_{t_{1}},x_{{t_{1}}+1},\ldots,x_{t_{2}}$ for *t*_1_≤*t*_2_. Given the HMM parameters, *θ*=(*A*,*O*,*π*), the likelihood of an observed sequence is:

$${\fontsize{9pt}{9.6pt}\selectfont{\begin{aligned} P(x_{1:T}|\theta) &= \sum_{h_{1},h_{2},\ldots,h_{T}}{P\left(x_{1:T},h_{1:T}|\theta\right)} & \\[-2pt] &= \textbf{1}_{K}^{T}{AO}_{x_{T}}\ldots {AO}_{x_{2}}{AO}_{x_{1}}\pi & \\ &= \textbf{1}_{K}^{T}B_{x_{T}}\ldots B_{x_{2}}B_{x_{1}}\pi \end{aligned}}} $$

where *h*_*t*_ in the first equation is the hidden state at the *t*th segment and the summation is taken over all possible sequences of hidden states. *O*_*i*_= diag(*O*[ *i*,1],*O*[ *i*,2],…,*O*[ *i*,*K*]), i.e., *O*_*i*_ is a matrix diagonalizing the *i*th row of *O* where *O*_*i*_[ *j*,*j*]=*O*[ *i*,*j*] for 1≤*i*≤*N* and 1≤*j*≤*K* and non-diagonal elements in *O*_*i*_ are zero. *B*_*i*_ is called the observable operator [63], defined as *B*_*i*_=*AO*_*i*_, and $\textbf {1}_{K}^{T}$ is a vector [ 1,1,…,1] of size *K*.

#### Estimating the hidden Markov model parameters using spectral learning

The original paper [33] does not attempt to estimate parameters directly as it can be unstable. We use a method adapted from [64] for inferring general phylogenetic tree models (which include HMMs as a special case) and improve the method in a deterministic and principled way using major observations (see further discussion in Additional file 1). Given all observed consecutive triples of observations in the genome, (*x*_*t*_,*x*_*t*+1_,*x*_*t*+2_), 1≤*t*≤*T*−2, the marginal probabilities of observing the counts of singletons, pairs and triples in the data (the moments in the method of moments) are defined in vector and matrix form as follows:

$${} \begin{aligned} P_{1}[\!i] &= \text{Pr}[\!x_{t} = i], \quad 1 \leq i \leq N \\ P_{2,1}[\!i,j] &= \text{Pr}[\!x_{t+1}=i,x_{t}=j], \quad 1 \leq i,j \leq N \\ P_{3,x,1}[\!i,j] &= \text{Pr}[\!x_{t+2}=i,x_{t+1}=x,x_{t}=j], \quad\! 1 \leq i,x,j \leq N \\ P_{3,1}[\!i,j] &= \sum_{1 \leq x \leq N}{P_{3,x,1}[\!i,j]} & \\ &= \text{Pr}[\!x_{t+2}=i,x_{t}=j], \quad 1 \leq i,j \leq N. \end{aligned} $$

Note that the counts of triples (the third moment) is actually a third-order tensor but for computational reasons we represent it by a collection of matrices indexed by the middle observation *x*, where each matrix corresponds to a slice through the tensor. Hsu et al. [33] showed that we can infer the HMM parameters from these marginal probabilities as follows. Let *U* be an *N*×*K* matrix of the top *K* left singular vectors (computed by the SVD) of *P*_3,1_. Intuitively, *U* is a surrogate for the observation matrix *O*.

We computed the following matrix *C*_*x*_ for each observation *x* by expressing the sample moments in terms of the parameters, *P*_3,*x*,1_=*OAO*_*x*_*A*diag(*π*)*O*^*T*^ and *P*_3,1_=*OAA*diag(*π*)*O*^*T*^,

(1) $${} C_{x} := \left(U^{T}P_{3,x,1}\right)\left(U^{T}P_{3,1}\right)^{+} = \left(U^{T}OA\right)O_{x}\left(U^{T}OA\right)^{-1}   $$

where *M*^+^ is the Moore–Penrose pseudoinverse of *M*. Since *O*_*x*_ is a diagonal matrix, it is easily seen that *U*^*T*^*OA* represents the eigenvectors of the matrix and the diagonal elements of *O*_*x*_ are exactly the eigenvalues. We will discuss how to compute the eigenvectors for epigenomic data below. For now, suppose that the eigenvectors, *U*^*T*^*OA*, are given. Then for each observation *x*,

(2) $${} \begin{aligned} \left(U^{T}OA\right)^{-1}C_{x}\left(U^{T}OA\right) &= \left(U^{T}OA\right)^{-1}\left(U^{T}OA\right)O_{x}\\ &\quad \times\left(\!U^{T}OA\!\right)^{-1}\left(U^{T}OA\right) = O_{x}. \end{aligned}   $$

Thus we can infer the emission matrix elements for *x* and the emission matrix *O* is inferred by combining all the *O*_*x*_’s. Note that we cannot infer the emission matrix elements directly from the eigenvalues computed from the above matrix *C*_*x*_ because we do not know the order of the eigenvalues. We circumvent this problem by utilizing that the eigenvectors are the same for all observations. Given the emission probabilities, the remaining parameters of the HMM, *π* and *A*, are easily computed by expressing the sample moments in terms of the parameters, *P*_1_=*Oπ* and *P*_2,1_=*OA*diag(*π*)*O*^*T*^, and the assumption that *A*,*O* and diag(*π*) are rank *K* (i.e. full-rank) as follows:

(3) $$\begin{array}{@{}rcl@{}} \pi &=& O^{+}P_{1}. \end{array} $$

(4) $$\begin{array}{@{}rcl@{}} A &=& \left(O^{+}O\right)A\left(\text{diag}(\pi)\text{diag}(\pi)^{-1}\right) \\ &=& \left(O^{+}O\right)A\text{diag}(\pi)\left(O^{+}O\right)^{T}\text{diag}(\pi)^{-1} \\ &=& O^{+}\left(OA\text{diag}(\pi)O^{T}\right)\left(O^{+}\right)^{T}\text{diag}(\pi)^{-1}  \\ &=& O^{+}P_{2,1}\left(O^{+}\right)^{T}\text{diag}(\pi)^{-1}.  \end{array} $$

The original algorithm of [33] assumed that we have access to many short samples from the HMM. When adapting the algorithm for a few long samples (e.g. chromosomes in the epigenomic data), we found that the distribution of initial observations was quite different from the distribution of all observations. Estimating the initial state distribution *π* from the distribution of all observations *P*_1_ introduces a significant amount of noise. Therefore, we modified *P*_1_ to $P^{\text {init}}_{1}$, which is the distribution of the first segment of all the chromosomes and we slightly modified Equation 3 as

(5) $$ \pi = O^{+}P^{\text{init}}_{1}.   $$

More significantly, we empirically found that most entries of *π* are close to zero and so taking the inverse of *π* in the calculation of *A* would introduce noise. Thus, we modified the computation of *A* to remove the dependence on *π* as follows:

(6) $${} {\fontsize{9.5pt}{9.6pt}\selectfont{\begin{aligned} A &= A\left(A\text{diag}(\pi)O^{T}\right)\left(A\text{diag}(\pi)O^{T}\right)^{+} \\ &= \left(\left(O^{+}O\right)A\left(A\text{diag}(\pi)O^{T}\right)\right)\left(\left(O^{+}O\right)\left(A\text{diag}(\pi)O^{T}\right)\right)^{+}\\ &= \left(O^{+}P_{3,1}\right)\left(O^{+}P_{2,1}\right)^{+}. \end{aligned}}}  $$

#### Computing the eigenvectors using major observations

It is not easy to compute the eigenvectors correctly because of noise in the data. If we compute *U*^*T*^*OA* from the matrix for each observation, we will generally get different *U*^*T*^*OA*’s for each observation in practice. Thus, we used a method based on our empirical observations about the epigenomic data.

For each state, we identified the major (i.e. most frequently occurring) observation in the genome segments labeled with that state by taking the corresponding singular vectors from the matrix *U* and using it to perform the eigen decomposition. Note that this approach differs from that of [65], which required a much stronger anchor word assumption, where for each chromatin state there is some epigenetic mark combination that occurs only in that state and none of the others. For each hidden state *i*, we picked the major observation *x*^′^=argmax_*x*_{*U*[ *x*,*i*]^2^}, analogously to a previous method for learning topic models from text documents [34]. Empirically we found that the major observation tends to appear in the chromatin state much more often than in the other chromatin states but we stress that this is not required by our method. Then we computed eigenvectors from $C_{x^{\prime }}\phantom {\dot {i}\!}$ and extracted a single eigenvector corresponding to the largest eigenvalue, which in practice was usually well separated from the other eigenvalues. We did this for all states separately and combined the eigenvectors into a single matrix.

In addition to picking a major observation for each state *i*, we also tried to compute the weighted summation of *C*_*x*_’s weighted by *U*[ *x*,*i*]^2^ for computing the eigenvectors. If the major observation makes *U*[ *x*,*i*]^2^ close to 1, the result of taking the sum will be very similar to our method of taking the max, which is what we indeed observed.



### Handling noisy data

Since the observation data are noisy, some numerical issues can occur when computing the SVD and eigen decomposition. Previously, [66] described implementation issues using spectral learning for a different latent variable model in natural language processing. We implemented several optimizations to solve the analogous issues for HMMs.

#### Handling negative probabilities

The estimated probabilities should be non-negative but the estimated parameters can have negative values because the signs can be flipped while performing the numerical computations. Therefore, we took the absolute value of the estimated parameters and normalized the parameters following [66].

#### Parameter adjustment

Although we estimated the HMM parameters using spectral learning (1), these estimates can sometimes be improved slightly by using the estimates from spectral learning as an initializer to the EM algorithm. We did not use this approach for the results reported in the main text but did so for the results with the Scripture binarization reported in Additional file 1 and this option is provided for users of the software.

#### Smoothing of observation matrices

Finally, although we did not use this optimization for the biological results reported here, our software allows for smoothing the observed pairs and triples matrices to address noise and data sparsity. Intuitively, our smoothing method is similar to adding pseudocounts to sparse data matrices except it uses the marginal frequencies of the observations instead of a uniform pseudocount. We provide this option for users who have noisier data than the ENCODE data.

### Data sets and experimental settings

For the epigenetic modification data, we used eight histone marks (H3K4me1, H3K4me2, H3K4me3, H3K9ac, H3K27ac, H3K27me3, H3K36me3 and H4K20me1) for eight ENCODE cell types (GM12878, H1-hESC, HMEC, HSMM, HUVEC, K562, NHEK and NHLF) [4] from the processed data from ENCODE [67]. For two additional ENCODE cell types, HeLa-S3 and HepG2, the processed data for the histone mark H3K4me1 were not available and so we used seven histone marks for those two cell types. We note that H3K4me2 can be used instead of H3K4me1 for identifying enhancers, so we expect all of the ten cell types we tested to give meaningful biological results. The processed ChIP-seq data from ENCODE were binarized following the Poisson binarization method of [7]. From the binarization, our final data set consisted of presence/absence calls for each histone mark for each segment, where the human reference genome was divided into 15,181,508 segments of size 200 bp. The observation space consisted of all non-zero combinations of epigenetic marks and in our case the number of possible observations was 2^8^=256. We fixed the number of chromatin states to 20 unless stated otherwise, following the suggestion of previous studies (e.g. [7]). This number of chromatin states is readily interpretable biologically and allows us to compare our annotations with previously published annotations.

For further biological analysis, we focused on three ENCODE Tier 1 cell types (GM12878, H1-hESC and K562) and we used external biological data sets to validate our inferred chromatin states. GM12878 is a B-lymphocyte cell line infected by the Epstein–Barr virus, H1-hESC is a human embryonic stem cell line and K562 is an erythrocytic leukemia cell line. We used active TSS data from Cap Analysis of Gene Expression (CAGE) and polyA RNA-seq data, both from ENCODE [68] (version 10, May 2012). RNA Polymerase II (Pol2), P300 ChIP-seq and DNase I hypersensitivity data were also used. We used long non-coding RNAs from [69], and conserved enhancer regions from the VISTA enhancer browser [70]. For distal P300 peaks, we removed peaks within ±2.5 kb of the above TSS data. Also, we used PRMs and DRMs derived from transcription-related factor binding data [43]. All of the data sets except the VISTA enhancer data were specific to the cell type that we used for training the HMM. We excluded genomic segments (approximately 0.3% of the genome) in the Duke excludable regions following [22].

### Gene ontology term analysis

We used the program GREAT [42] to identify over-represented GO terms for regulatory elements. For promoters, we tested segments in a promoter state against all segments in all promoter states as background (parameters: proximal was 1 kb upstream and 1 kb downstream and no distal). For enhancers, we tested segments with distal P300 signal in an enhancer state against all segments with distal P300 signal in all enhancer states as background (parameters as default: proximal was 5 kb upstream and 1 kb downstream and distal was up to 1,000 kb).

### Conservation analysis

We quantified the levels of selective constraint acting on the Spectacle chromatin states at the population genetic and cross-species levels. To analyze population genetic levels of constraint, we computed the MAFs of all SNPs from the European populations in the 1000 Genomes Project [71], processed as previously described [72]. We chose to use the MAF statistic instead of rooting the SNPs (e.g. with the chimpanzee allele) to avoid complications with multiple mutations at highly mutable sites (e.g. CpG sites) and because the extent of positive selection in humans is expected to be low due to the low effective population size. Thus the MAF is expected to be a reasonable indicator of the strength of negative selection on the SNPs. To minimize the effects of very rare variations, which are likely to reflect primarily the nucleotide mutational process, we removed SNPs with MAF less than 0.01, leaving 16,455,987 SNPs. For each chromatin state, we quantified the level of selective constraint by computing the fraction of low-frequency SNPs in that chromatin state, specifically those with MAF less than 0.1. We compared this with a background set of SNPs consisting of the 11,015,766 SNPs not contained in genes annotated by RefSeq, following [72].

To quantify the level of evolutionary conservation across species, we downloaded phastCons scores for the primate subset of the 46 vertebrate species from the UCSC Genome Browser (phastCons46way.primates) [73]. For each segment in the genome, we computed the mean of phastCons scores of the 200 bases for a conservation score of the segment. For each chromatin state, we quantified the cross-species level of selective constraint by computing the mean of phastCons scores of all bases for all segments in that chromatin state.

## Additional file

Additional file 1
**Supplementary note.** Supplementary results, figures and tables.

## Abbreviations

Art: artificial
bp: base pair
ChIP-seq: chromatin immunoprecipitation sequencing
CpGI: CpG island
DRM: gene-distal regulatory module
EM: expectation-maximization
Enh: strong enhancer
EnhP: poised enhancer
EnhW: weak enhancer
GO: gene ontology
GWAS: genome-wide association study
HMM: hidden Markov model
kb: kilobase
MAF: minor allele frequency
Pol2: RNA polymerase II binding
PRM: promoter-proximal regulatory module
Prom: strong promoter
PromF: promoter flanking
PromP: poised promoter
PromW: weak promoter
Repr: repressed region
SNP: single nucleotide polymorphism
SOM: self-organizing map
SVD: singular value decomposition
TSS: transcription start site
Txn: transcribed region
UTR: untranslated region
