# Supplementary material for: Spectacle: fast chromatin state annotation using spectral learning
Source: Genome Biol. 2015 Feb 12;16(1):33. doi: 10.1186/s13059-015-0598-0 (PMC4355146; doi:10.1186/s13059-015-0598-0)
Supplement: Additional file 1 — Supplementary note. Supplementary results, figures and tables. [file 13059_2015_598_MOESM1_ESM.pdf]

1 Overview of our spectral learning approach

Spectral learning is a novel approach to parameter estimation for latent variable models (e.g. Hidden Markov Models, topic models, etc.) that relies on a Methods of Moments approach instead of Maximum Likelihood. In the Method of Moments approach, we express various expected moments of the model (e.g. mean, covariance etc.) as functions of the parameters, set the expected moments equal to the sample moments estimated from the data, and solve the resulting equations for the parameters. Spectral learning specializes this approach for latent variable models by making an additional mild assumption that various parameter matrices are full-rank. For example, in the case of HMMs, spectral learning is provably correct (i.e. it recovers the true parameters of the model) assuming that the transition and emission matrices of the HMM have rank equal to the number of chromatin states and that the initial state vector is strictly greater than 0 in all coordinates. One can also prove polynomial sample complexity bounds for the method. These assumptions are considered mild because of the general view that learning is not meaningful if the assumption is false – for example, if there is one emission probability vector for a chromatin state that is a linear combination of two others, or even identical to another chromatin state, that would be generally considered a degenerate model. In practical applications, spectral learning can be much faster than the EM algorithm since the main computation is simply to compute the singular value decomposition (SVD) of the second moment matrix. Unlike EM, spectral learning does not suffer from local optima issues and does not depend on the parameter initialization.

There are many open theoretical issues in spectral learning that the machine learning community is currently working on. For example, the problem of how to best include regularization into the spectral learning framework is a major open question. Beyond these theoretical problems, not many practical attempts have been made to implement and test this class of methods in any applied area. There are a few implementations of spectral learning in the natural language processing and computer vision communities, but these data types have different characteristics to genomics data. For example, in natural language processing, one often has access to many short samples whereas in genomics we have one very long sample (i.e. the genome). In the context of computational biology, to the best of our knowledge there are only two previous implementations which were cited in the Discussion section of the main manuscript. However, both of them were for very specific applications, one as a string kernel method within a discriminative classifier and one for contrasting a foreground and background data set in cases where this distinction makes biological sense.

The overall method of Spectacle is based on the method of [1] who learned the HMM parameters only up to an unknown invertible transformation. The transformed parameters are still useful for computing the likelihood of the data and predicting future observations (it is also called the "Observable Operator Model") but cannot be used to annotate the hidden chromatin states. Mossel and Roch showed how to infer the HMM parameters explicitly for the special case where the state and observation spaces have the same dimension [2]. [1] extended their method to more general HMMs. However, the published method can be unstable if the eigenvalues corresponding to the chromatin states are not well separated and the method relies

on injecting noise into the eigenvector computation to spread the eigenvalues [1]. In this work we have improved the method in a deterministic and principled way to infer the (untransformed) HMM parameters by using a single major observation for each chromatin state to compute the eigenvectors. In addition, [1] assumed that we have access to many short, independent samples from the HMM and we have modified their method to our application where we have a few long samples from the HMM by rewriting the formula for the transition matrix parameters in a form that does not depend on the initial state probability distribution. Finally, we have contributed empirically developed solutions to deal with noise for our application of chromatin state annotation. In our experiments, we made a novel observation that spectral learning tends to be more robust on class imbalanced data than the maximum likelihood approach. We believe this property is of significant interest for follow-up theoretical studies.

2 Comparison of the time complexity of spectral learning and the EM algorithm

The training time of one iteration of EM is linear in the number of genomic segments and the number of chromatin mark combinations, and quadratic in the number of chromatin states. On the other hand, the training time of spectral learning is dominated by the computation time of the SVD which is cubic in the number of observations if all singular values are computed exactly (this is the running time of a practical implementation - theoretically the cubic dependence can be improved to the exponent of matrix multiplication [3] which is currently < 2.373 [4]). In fact, the practical running time is significantly faster because we only need to compute the top K singular vectors corresponding to the K chromatin states and typically $K << N$.

Conversely, the training time of spectral learning depends exponentially on the number of histone marks if there is no redundancy between histone marks and all possible combinations of histone marks exist in the sample data. The total number of known histone marks is currently over 130 [5] but in practice the number of histone marks assayed in a single cell type is currently much smaller due to the expense and difficulty of the experiments. For example, there are less than 12 and 30 epigenetic marks in the ENCODE and Roadmap Epigenomics data, respectively, and there are less than 8 histone mark data for the majority of cell types. Furthermore, in practice, the number of observed combinations of chromatin marks is actually far smaller than the theoretically maximum number of combinations of chromatin marks, growing only as a small polynomial in the number of chromatin marks as opposed to exponential. Thus standard sparse linear algebra approaches are very efficient when applying spectral learning in the context of chromatin state annotation (see Main Text). Also, from a biological standpoint it is important in our view to study all possible combinations of chromatin marks to discover any possible interactions among the marks, rather than assuming that the marks are independent, as in other methods.

2.1 Analysis of the number of chromatin mark combinations

To analyze the actual number of combinations of chromatin marks that appear in real human epigenomics data, we used the following 16 chromatin marks in GM12878 – CTCF, H2AZ, H3K4me1/2/3, H3K9ac, H3K9me3, H3K27ac, H3K27me3, H3K36me3, H3K79me2, H4K20me1, P300, Pol2, DNase 1 hypersensitive sites and FAIRE. We found that the number of combinations of chromatin marks was a low-order polynomial (degree 1 to 2) of the number of marks (Figure S1). The numbers of pairs and triples of combinations of chromatin marks were also low-order polynomials in the number of chromatin marks (Figures S2,S3).

2.2 Runtime of Spectacle with larger numbers of epigenetic marks

In order to run Spectacle with a large number of chromatin marks, we used the version of our method implemented in Python using the SciPy sparse matrix library. We ran Spectacle and ChromHMM using 29 chromatin marks for embryonic stem cell H9 (new EID E008) from the Roadmap Epigenomics project. In theory, there can be up to 2^{29} combinations of chromatin marks, but in fact we observed that there were only 186,814 (∼0.03%) combinations appearing in the genome. After eliminating singleton combinations that are likely due to noise, there were only 56,750 (∼0.01%) combinations that appeared at least twice in the genome. Spectacle took ∼43.2 min after removing singleton combinations. On the other hand, it took ∼8.1 hr to run ChromHMM for 200 iterations without reaching convergence. We also note again that Spectacle considers all possible combinations of epigenetic marks while ChromHMM makes a conditional independence assumption between epigenetic marks.

3 Performance of spectral learning on a data set with more balanced classes

We tested Spectacle on a different binarization of the ENCODE ChIP-seq reads using the Scripture program [6]. Scripture produces broad peak calls for ChIP-seq reads, which is most appropriate for some histone marks such as H3K36me3 and H3K9me3 [7]. For most other known histone marks, sharp peak calls are more appropriate. Consistent with this, the Scripture peak calls covered 81-97% of the peaks called by the Poisson binarization method and we expect that the additional Scripture peak calls likely include some false positive peaks. Thus, the Scripture data is noisier and more balanced between different classes (∼52% of the genome was in the null state in Table 1 instead of ∼90%). Consistent with our observations about class imbalance, our experiments on the Scripture data showed that the maximum likelihood criterion is a good criterion in the class balanced case in the sense that improvement in chromatin state annotation (as judged by overlap with biological features) corresponded to higher likelihoods. On this data set, we thus tested the possibility of using Spectacle as an initializer to the EM algorithm, which is another common use of Method of Moments estimators (e.g. [8, 9]).

We first benchmarked the performance of the EM algorithm with either random parameter initialization or a published heuristic initializer. For 15 chromatin states, we ran the EM algorithm to convergence for 10 random parameter initializations

for the GM12878 cell line. The log-likelihood varied from $-8.33E6$ to $-1.11E7$ (average: $-9.51E6$, std. dev.: $7.67E5$) and the number of iterations needed for the likelihood to converge varied from 16 to 200. We found that the highest log-likelihood from the random initializations was lower than that of the heuristic initialization method, *information*, which was $-7.81E6$. We thus used the information heuristic as a baseline for further comparisons.

We compared Spectacle with a few (up to 5) iterations of EM with ChromHMM with the information initializer in terms of the likelihood of the data for an ENCODE Tier 1 cell line GM12878. For almost all numbers of states we tested, Spectacle had a higher likelihood than ChromHMM with the information initializer (Table S1). For the case of 100 states, the difference in the performance of the two methods was rather large and the likelihood found by ChromHMM was lower than that for fewer chromatin states, suggesting that the EM algorithm found a local optimum for this particular number of states. Since ChromHMM took 20-200 EM iterations to converge to a local optimum in the likelihood, we next ran EM for only five iterations to make its runtime comparable to the spectral learning approach. In this case we found that spectral learning had a higher likelihood than ChromHMM with the information initializer for all numbers of states tested. Taken together, these results show that when the run times of the two methods were constrained to be similar, spectral learning always outperformed EM, and even when spectral learning took a much shorter runtime, it still generally performed better than EM as judged by the likelihood.

We then compared both methods over multiple cell types to assess the performance of Spectacle over a range of data sets. We examined ten cell types including GM12878 and ran spectral learning and ChromHMM with 15 hidden chromatin states. Spectral learning found higher likelihoods for 8 out of 10 cell types while also achieving a 2.6-12.6 fold (in CPU time) and 2.6-13.4 fold (in number of iterations) faster training time (Table S2). For 20 chromatin states, Spectral learning found higher likelihoods for 9 out of 10 cell types while also achieving a 2.6-12.6 fold (in CPU time) and 2.6-12.6 fold (in number of iterations) faster training time (Table S3). Finally we verified that the higher likelihoods indeed corresponded to improved precision-recall accuracy on a number of biological features (Figures S4-S13).

Taken together, from our testing on the Scripture data set, we found that Maximum Likelihood was a good criteria for class balanced epigenomic data. In this case Spectacle was useful as a parameter initialization method to the EM or other local search algorithm, in which case it improved on the run time, likelihood and precision-recall accuracy compared to the published "information" heuristic initializer. Although this approach does not appear to be required on current human or mammalian epigenomic data sets, we note that non-vertebrate species with more compact genomes are likely to have more class balanced epigenomic data sets because they have a higher density of genes and regulatory elements compared with non-regulatory non-coding regions so this approach may be useful for those data sets.

4 Comparison of Spectacle with Segway and TreeHMM

To compare the results of Spectacle with Segway, we downloaded the chromatin mark datasets used in Hoffman et al [10] and the genome segmentations produced by Segway when run on the datasets. We ran Spectacle with 25 chromatin states on the same datasets (Figures S20,S21). The fraction of the genome that did not have any signal was ∼81%. Thus, it was still a class-imbalanced data set although the number of epigenetic marks (14 marks) was higher than the data set used in the main text (eight marks). Segway assigned 12 of the 25 chromatin states (i.e. about half of the chromatin states) as inactive states (Quies, Low, Repr), three states as promoter states and four states as enhancer states. On the other hand, Spectacle assigned only four states as inactive states, four states as promoter states, and nine states as stong and weak enhancer states. Consistent with our results with 20 chromatin states in the main text, GWAS SNPs from 62 out of 78 phenotypes and in particular 12 out of 14 autoimmune diseases were found to be most enriched in one of the strong enhancer states for Spectacle (Table S6). Thus, taken together, these results were consistent with our observations about the performance of spectral learning vs. the EM algorithm on class imbalanced data sets. Since Segway uses a graphical model quite similar to a standard Hidden Markov Model and uses a Maximum Likelihood framework for inference, we expected its performance to be more similar to that of ChromHMM than Spectacle.

We also compared our results with TreeHMM. In Figure 4 of the TreeHMM paper, it can be seen that 4 of the 18 chromatin states were assigned as “low signal” states and an additional state was assigned as a repetitive state. Thus we believe that the same issues of overfitting to the background null state also affect TreeHMM, consistent with the fact that TreeHMM also uses maximum likelihood and an approximation to the EM algorithm for estimating the model parameters. Due to the higher number of parameters devoted to the null states, TreeHMM produced fewer promoter and enhancer states than Spectacle (Figures 2,S14,S16). It is not possible to directly compare with the solution of Spectacle with 20 chromatin states because TreeHMM learned slightly fewer chromatin states (18) and it used the CTCF signal which was not included in our dataset. However, Spectacle found a higher number of strong enhancer sub-types that did not exist in the TreeHMM solution (e.g. state 7, 17 and 20 in GM12878 and state 15 and 20 in K562). Note that state 20 in GM12878 and state 20 in K562 were the enhancer sub-types with the highest GWAS SNP enrichment in our analyses in the main text. TreeHMM also produces an additional type of output, namely comparative chromatin state annotations between multiple cell types, as described in the main text. This output is not directly comparable to the output of our combined cell type analysis because TreeHMM only learns a single set of chromatin states for all the cell types whereas we learn combined chromatin states that can differ for each cell type. Also, TreeHMM produces different genomic segmentations for each cell type and comparison of specific chromatin states, such as enhancers, must be done as a post-processing step. In contrast, our approach produces a single consistent genomic segmentation of all cell types. Nonetheless, both methods are successful at revealing cell-type specific enhancers.

5 Cross-validation analysis of the model

Spectacle considers all possible combinations of chromatin marks intead of assuming that they are conditionally independent of each other given the hidden state. In theory, this can increase the number of parameters exponentially. Fortunately, the actual number of combinations of chromatin marks that appear in the real human epigenomics data is much less than the possible number of combinations (refer to Supplementary Note 2). Still, the number of parameters is much higher than that in the model with the conditional independence assumption. Overfitting can be an issue if the model complexity is high and the dataset is relatively small. We addressed this issue by doing a cross-validation analysis. More specifically, for this analysis we used the combined dataset for three cell types with 23 chromatin marks, the same dataset used in our manuscript (Results section 2.6). We divided the dataset into training and test data sets 23 different times, each time holding out one chromosome as test data. We trained our model and estimated parameters on the training data by using our method Spectacle, predicted chromatin states on a test data from the estimated parameters by using the posterior decoding algorithm, and tested the predicted chromatin states by using independent validation datasets such as active TSSs and active enhancers. We found that precisions and recalls for predicting TSSs and enhancers from the cross-validation analysis were almost identical to those from the case where both training and test data are the whole dataset (Table S10). This demonstrates that overfitting is not a major concern in our data.

Author details

References

1. Hsu, D., Kakade, S.M., Zhang, T.: A spectral algorithm for learning hidden Markov models. Journal of Computer and System Sciences **78**, 1460–1480 (2012)
2. Mossel, E., Roch, S.: Learning nonsingular phylogenies and hidden Markov models. The Annals of Applied Probability **16**(2), 583–614 (2006)
3. Demmel, J., Dumitriu, I., Holtz, O.: Fast linear algebra is stable. Journal Numerische Mathematik **108**(1), 59–91 (2007)
4. Williams, V.V.: Multiplying matrices faster than Coppersmith-Winograd. In: Symposium on the Theory of Computing ($STOC$), pp. 887–898 (2012)
5. Rivera, C.M., Ren, B.: Mapping human epigenomes. Cell **155**(1), 39–55 (2013)
6. Guttman, M., Garber, M., Levin, J.Z., Donaghey, J., Robinson, J., Adiconis, X., Fan, L., Koziol, M.J., Gnirke, A., Nusbaum, C., Rinn, J.L., Lander, E.S., Regev, A.: Ab initio reconstruction of cell type-specific transcriptomes in mouse reveals the conserved multi-exonic structure of lincRNAs. Nature Biotechnology **28**(5), 503–510 (2010)
7. Landt, S.G., Marinov, G.K., Kundaje, A., Kheradpour, P., Pauli, F., Batzoglou, S., Bernstein, B.E., Bickel, P., Brown, J.B., Cayting, P., Chen, Y., DeSalvo, G., Epstein, C., Fisher-Aylor, K.I., Euskirchen, G., Gerstein, M., Gertz, J., Hartemink, A.J., Hoffman, M.M., Iyer, V.R., Jung, Y.L., Karmakar, S., Kellis, M., Kharchenko, P.V., Li, Q., Liu, T., Liu, X.S., Ma, L., Milosavljevic, A., Myers, R.M., Park, P.J., Pazin, M.J., Perry, M.D., Raha, D., Reddy, T.E., Rozowsky, J., Shoresh, N., Sidow, A., Slattery, M., Stamatoyannopoulos, J.A., Tolstorukov, M.Y., White, K.P., Xi, S., Farnham, P.J., Lieb, J.D., Wold, B.J., Snyder, M.: ChIP-seq guidelines and practices of the ENCODE and modENCODE consortia. Genome Research **22**, 1813–1831 (2012)
8. Ye, X., Yu, Y.-K., Altschul, S.F.: On the inference of Dirichlet mixture priors for protein sequence comparison. Journal of computational biology **18**(8), 941–954 (2011)
9. Chaganty, A.T., Liang, P.: Spectral experts for estimating mixtures of linear regressions. In: The 30th International Conference on Machine Learning ($ICML$), vol. 28, pp. 1040–1048 (2013)
10. Hoffman, M.M., Ernst, J., Wilder, S.P., Kundaje, A., Harris, R.S., Libbrecht, M., Giardine, B., Ellenbogen, P.M., Bilmes, J.A., Birney, E., Hardison, R.C., Dunham, I., Kellis, M., Noble, W.S.: Integrative annotation of chromatin elements from ENCODE data. Nucleic Acids Research **41**(2), 827–841 (2013)

Table S1 Comparison of the log-likelihood of Spectacle with five EM iterations and ChromHMM when run up to convergence while varying the number of chromatin states. For each number of chromatin states, the method with higher log-likelihood appears in bold. Within the parentheses is the log-likelihood ratio between ChromHMM and Spectaclewhen both methods are run with only five EM iterations. This is for the GM12878 cell type with Scripture peak calls.

Num of states	ChromHMM	Spectacle	Log-likelihood ratio ($\times 10^4$)
5	-1.74e+07	**-1.65e+07**	82.9
10	-9.32e+06	**-9.03e+06**	29.5
15	-7.81e+06	**-7.17e+06**	64.2
20	-6.31e+06	**-6.29e+06**	2.1
25	**-5.67e+06**	-5.71e+06	-3.8 (6.8)
30	-5.29e+06	**-5.26e+06**	2.6
35	-5.06e+06	**-4.93e+06**	12.9
40	-4.75e+06	**-4.67e+06**	7.7
45	**-4.46e+06**	-4.47e+06	-1.3 (4.2)
50	**-4.24e+06**	-4.32e+06	-8.0 (4.1)
55	-4.09e+06	**-4.05e+06**	3.8
60	-3.931e+06	**-3.87e+06**	5.9
65	-3.80e+06	**-3.75e+06**	5.0
70	-3.70e+06	**-3.64e+06**	6.0
75	-3.60e+06	**-3.56e+06**	3.9
80	-3.53e+06	**-3.50e+06**	2.5
85	-3.45e+06	**-3.42e+06**	3.1
90	-3.40e+06	**-3.36e+06**	4.5
95	-3.34e+06	**-3.29e+06**	5.2
100	-4.58e+06	**-3.23e+06**	134.7
105	-3.23e+06	**-3.18e+06**	4.9

Table S2 The log-likelihood of Spectacle with five EM iterations and ChromHMM when run up to convergence for ten cell types for 15 states with Scripture peak calls. The method with higher log-likelihood is made bold. Within the parentheses is the log-likelihood ratio between ChromHMM and Spectacle when both of the methods are run with only five EM iterations.

Cell type	ChromHMM	Spectacle	Log-likelihood ratio ($\times 10^4$)
GM12878	-7.81e+06	**-7.17e+06**	64.3
H1-hESC	-1.15e+07	**-1.14e+07**	7.0
HeLa-S3	**-9.67e+06**	-9.99e+06	-31.4 (-16.1)
HepG2	-7.99e+06	**-7.88e+06**	11.1
HMEC	-7.87e+06	**-7.57e+06**	30.0
HSMM	-8.71e+06	**-8.29e+06**	42.3
HUVEC	**-6.44e+06**	-6.61e+06	-17.2 (-14.6)
K562	-7.64e+06	**-7.50e+06**	13.3
NHEK	-7.82e+06	**-7.74e+06**	8.2
NHLF	-9.11e+06	**-8.98e+06**	13.3

Table S3 The log-likelihood of Spectacle with five EM iterations and ChromHMM when run up to convergence for ten cell types for 20 states for Scripture peak calls. The method with higher log-likelihood is made bold. Within the parentheses is the log-likelihood ratio between ChromHMM and Spectacle when both of the methods are run with only five EM iterations.

Cell type	ChromHMM	Spectacle	Log-likelihood ratio ($\times 10^4$)
GM12878	-6.31e+06	**-6.29e+06**	2.1
H1-hESC	-9.90e+06	**-9.77e+06**	13.4
HeLa-S3	-8.48e+06	**-8.30e+06**	17.7
HepG2	-6.87e+06	**-6.64e+06**	22.7
HMEC	-6.70e+06	**-6.52e+06**	18.3
HSMM	**-7.28e+06**	-7.30e+06	-1.9 (18.8)
HUVEC	-5.77e+06	**-5.58e+06**	18.8
K562	-6.81e+06	**-6.67e+06**	14.4
NHEK	-6.78e+06	**-6.73e+06**	4.3
NHLF	-7.96e+06	**-7.60e+06**	36.3

Table S4 Number of phenotypes whose SNPs were most highly enriched in a strong enhancer state, and number of all non-coding SNPs associated with the phenotypes in H1-hESC

Method	State	Num phenotypes	Num associated SNPs
Spectacle	11	5	627
ChromHMM	11	17	596

Table S5 Number of phenotypes whose SNPs were most highly enriched in a strong enhancer state, number of all non-coding SNPs associated with the phenotypes, and number of erythrocyte related phenotypes among the most highly enriched phenotypes in K562

Method	State	Num phenotypes	Num associated SNPs	Num erythrocyte
Spectacle	7	13	436	0
Spectacle	8	16	418	0
Spectacle	15	20	1143	0
Spectacle	16	13	785	0
Spectacle	20	24	1614	5
ChromHMM	7	2	34	1
ChromHMM	8	5	66	0
ChromHMM	15	5	309	0

Table S6 Number of phenotypes whose SNPs were most highly enriched in a strong enhancer state predicted by either Spectacle or Segway, number of all non-coding SNPs associated with the phenotypes, and number of autoimmune disease related phenotypes among the most highly enriched phenotypes in GM12878 for the Hoffman et al [10] dataset

Method	State	Num phenotypes	Num associated SNPs	Num autoimmune
Spectacle	14	16	912	8
Spectacle	17	10	208	2
Spectacle	18	18	552	2
Spectacle	22	13	259	0
Spectacle	24	5	71	0
Segway	16	1	17	0
Segway	25	15	784	2

Table S7 The fraction of SNPs in each chromatin state in Spectacle with minor allele frequencies (MAFs) less than 0.1 is shown. Chromatin states are sorted in a decreasing order. The fraction of SNPs in the background with MAF less than 0.1 is .5181.

GM12878			H1-hESC			K562		
State	Annot.	MAF< 0.1	State	Annot.	MAF< 0.1	State	Annot.	MAF< 0.1
13	Prom	.5722	17	Art	.5899	19	PromF	.5999
5	Prom	.5658	7	Prom	.5872	6	Prom	.5747
2	Txn	.5636	14	Txn	.5712	15	Enh	.5670
15	Txn	.5571	8	Prom	.5711	2	Txn	.5638
19	Prom	.5547	3	Txn	.5637	12	Prom	.5576
12	EnhW	.5539	12	EnhP	.5633	20	Enh	.5542
14	PromW	.5538	4	Repr	.5601	14	EnhW	.5540
11	PromW	.5510	20	EnhP	.5586	9	EnhW	.5533
10	EnhW	.5506	18	PromP	.5571	10	EnhW	.5525
18	PromP	.5426	15	PromW	.5560	11	PromW	.5498
17	Enh	.5415	13	EnhP	.5534	7	Enh	.5461
9	Enh	.5414	10	EnhW	.5518	13	Prom	.5414
8	Enh	.5354	19	EnhP	.5472	17	EnhW	.5388
6	EnhW	.5348	11	Enh	.5444	5	Repr	.5372
7	Enh	.5283	16	EnhP	.5414	4	EnhW	.5310
4	Repr	.5266	6	EnhW	.5334	8	Enh	.5303
20	Enh	.5264	2	EnhW	.5271	16	Enh	.5288
16	EnhW	.5251	5	Repr	.5246	1	Repr	.5216
3	Repr	.5240	9	Repr	.5241	3	Repr	.5105
1	Repr	.5212	1	Repr	.5220	18	Art	.4944

Table S8 The mean of phastCons scores in each chromatin state in Spectacle is shown. Chromatin states are sorted in a decreasing order.

GM12878			H1-hESC			K562		
State	Annot.	PhastCons	State	Annot.	PhastCons	State	Annot.	PhastCons
13	Prom	.2427	14	Txn	.2226	6	Prom	.1734
19	Prom	.1536	12	EnhP	.2106	19	PromF	.1726
11	PromW	.1488	15	PromW	.2032	15	Enh	.1497
5	Prom	.1460	20	EnhP	.2031	12	Prom	.1436
17	Enh	.1446	13	EnhP	.1992	13	Prom	.1427
2	Txn	.1319	7	Prom	.1981	20	Enh	.1351
10	EnhW	.1287	19	EnhP	.1896	2	Txn	.1334
14	PromW	.1279	18	PromP	.1728	9	EnhW	.1233
12	EnhW	.1262	8	Prom	.1693	14	EnhW	.1194
3	Repr	.1223	11	Enh	.1623	7	Enh	.1165
18	PromP	.1085	3	Txn	.1602	11	PromW	.1094
9	Enh	.1046	16	EnhP	.1474	16	Enh	.1037
8	Enh	.1038	6	EnhW	.1411	3	Repr	.0916
6	EnhW	.1003	10	EnhW	.1273	4	EnhW	.0910
16	EnhW	.0960	2	EnhW	.1176	8	Enh	.0903
1	Repr	.0865	5	Repr	.1013	10	EnhW	.0898
4	Repr	.0862	9	Repr	.0897	1	Repr	.0876
20	Enh	.0859	4	Repr	.0885	17	EnhW	.0873
15	Txn	.0858	1	Repr	.0859	18	Art	.0870
7	Enh	.0844	17	Art	.0709	5	Repr	.0784

Table S9 Number of phenotypes whose SNPs were most highly enriched in a strong cell type-specific enhancer state, number of all non-coding SNPs associated with the phenotypes, and number of autoimmune disease-related phenotypes or liver-related phenotypes among the most highly enriched phenotypes in Spectacle states for the cell type combined dataset

State	Cell type	Num phenotypes	Num associated SNPs	Num autoimmune	Num liver
14	GM12878	8	117	0	0
20	GM12878	5	65	2	0
26	GM12878	7	167	0	0
34	GM12878	3	39	0	0
39	GM12878	28	1432	11	0
44	GM12878	9	114	0	0
47	GM12878	30	844	1	0
48	GM12878	6	129	0	0
28	H1-hESC	5	67	0	1
38	H1-hESC	5	242	0	0
15	HepG2	7	140	0	0
36	HepG2	10	428	0	0
46	HepG2	9	231	0	0

Table S10 Precision and recall for predicting external validation datasets. Validation datasets are active Gencode TSS or distal P300 for three cell types. "Whole Genome" denotes that parameters were trained on the whole genome. CV (cross-validation) denotes that parameters were trained on all of the genome except for one chromosome and chromatin states for the held out chromosome were predicted by using the trained parameters. In this way, chromatin states for the whole genome were predicted.

Validation dataset	Cell type	Whole Genome	CV
TSS	GM12878	0.048, 0.920	0.048, 0.919
TSS	H1-hESC	0.054, 0.904	0.053, 0.904
TSS	HepG	0.062, 0.916	0.061, 0.918
distal P300	GM12878	0.087, 0.465	0.086, 0.462
distal P300	H1-hESC	0.092, 0.303	0.092, 0.302
distal P300	HepG	0.249, 0.421	0.249, 0.421

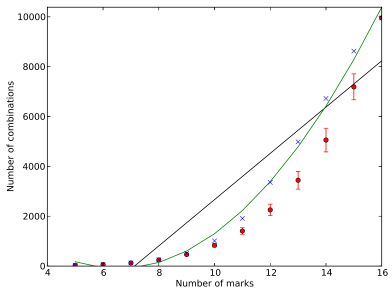

Figure S1 Growth in the number of combinations of marks (Y axis) as a function of the number of marks (X axis). Each red mark, its error bar, and blue mark for x marks is the average, standard deviation, and maximum, repectively, of the numbers of combinations of marks when choosing x out of the 16 marks. Black and green line is a polynomial of degree 1 and 2, respectively, that fits the blue marks.

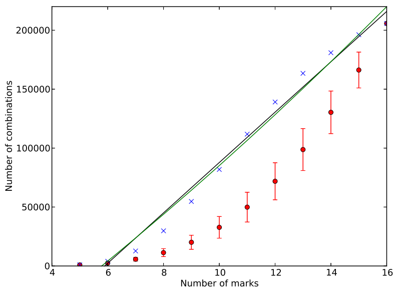

Figure S2 Growth in the number of pairs of combinations of marks (Y axis) as a function of the number of marks (X axis). Refer to caption of Figure S1.

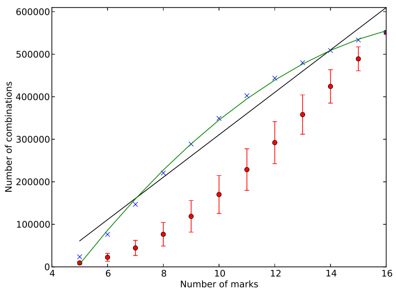

Figure S3 Growth in the number of triples of combinations of marks (Y axis) as a function of the number of marks (X axis). Refer to caption of Figure S1.

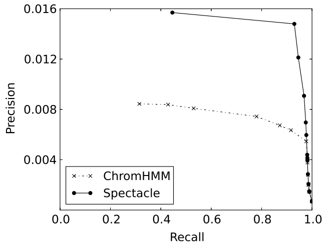

Figure S4 Precision-Recall curve for prediction of active TSS regions when the number of states is 15.

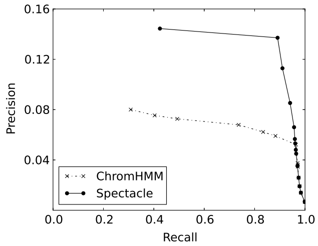

Figure S5 Precision-Recall curve for prediction of active TSS plus flanking regions (within ±1kb) when the number of states is 15.

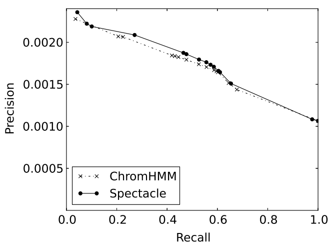

Figure S6 Precision-Recall curve for prediction of VISTA enhancers when the number of states is 15.

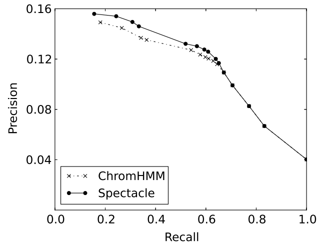

Figure S7 Precision-Recall curve for prediction of transcribed regions when the number of states is 15.

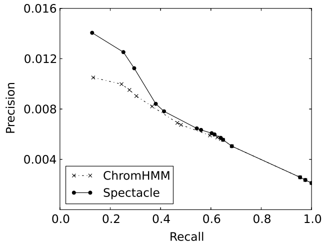

Figure S8 Precision-Recall curve for prediction of lincRNAs when the number of states is 15.

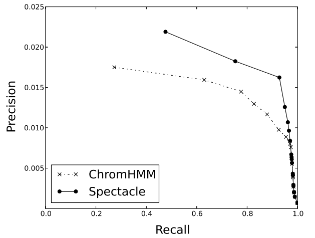

Figure S9 Precision-Recall curve for prediction of active TSS regions when the number of states is 20.

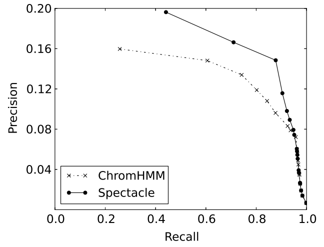

Figure S10 Precision-Recall curve for prediction of active TSS plus flanking regions (within $\pm$1kb) when the number of states is 20.

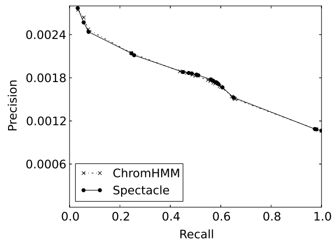

Figure S11 Precision-Recall curve for prediction of VISTA enhancers when the number of states is 20.

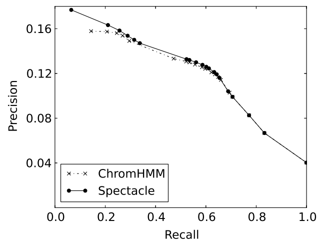

Figure S12 Precision-Recall curve for prediction of transcribed regions when the number of states is 20.

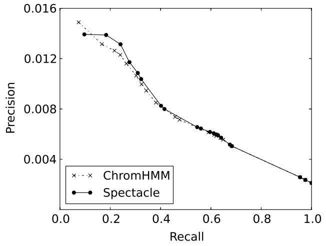

Figure S13 Precision-Recall curve for prediction of lincRNAs when the number of states is 20.

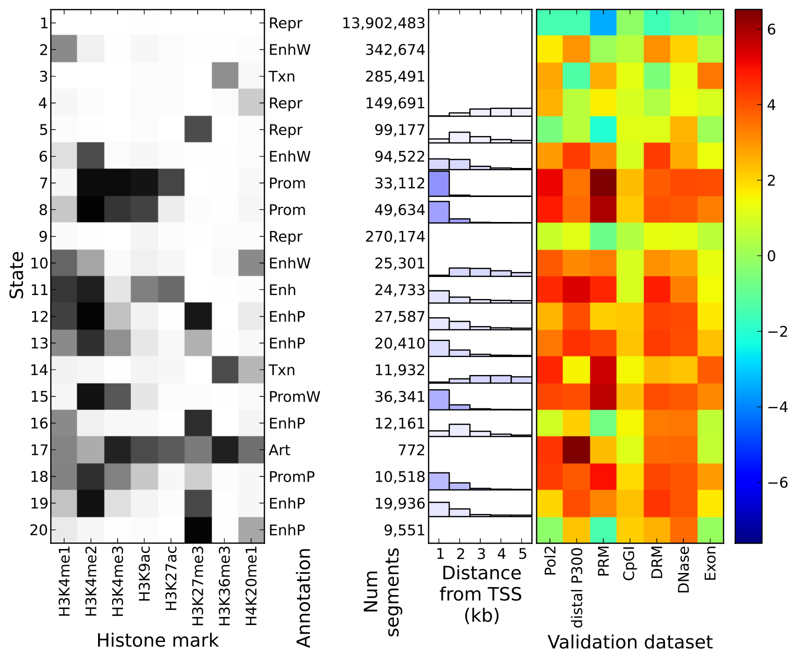

Figure S14 Spectacle emission matrix and state annotation for cell line H1-hESC for 20 chromatin states. Refer to caption of Figure 1 in the main text.

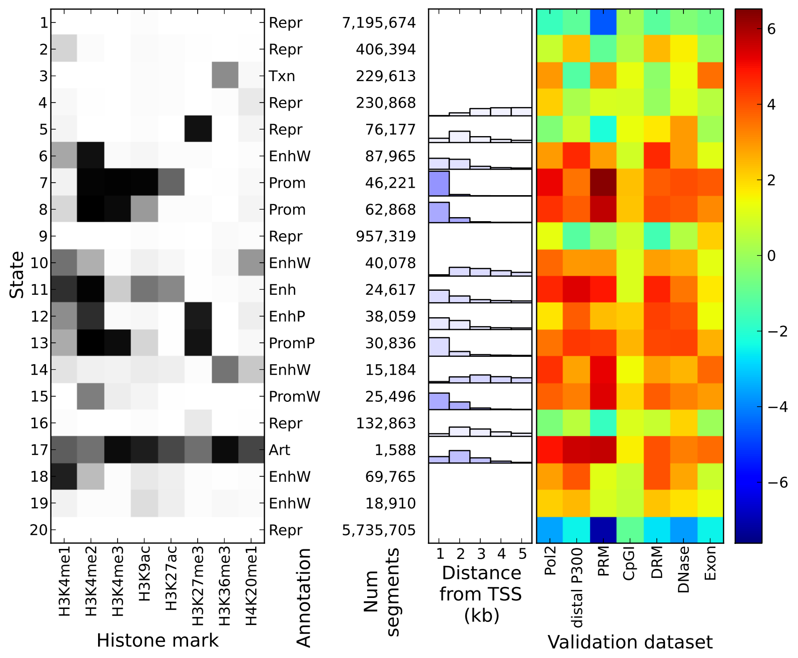

Figure S15 ChromHMM emission matrix and state annotation for cell line H1-hESC for 20 chromatin states. Refer to caption of Figure 1 in the main text.

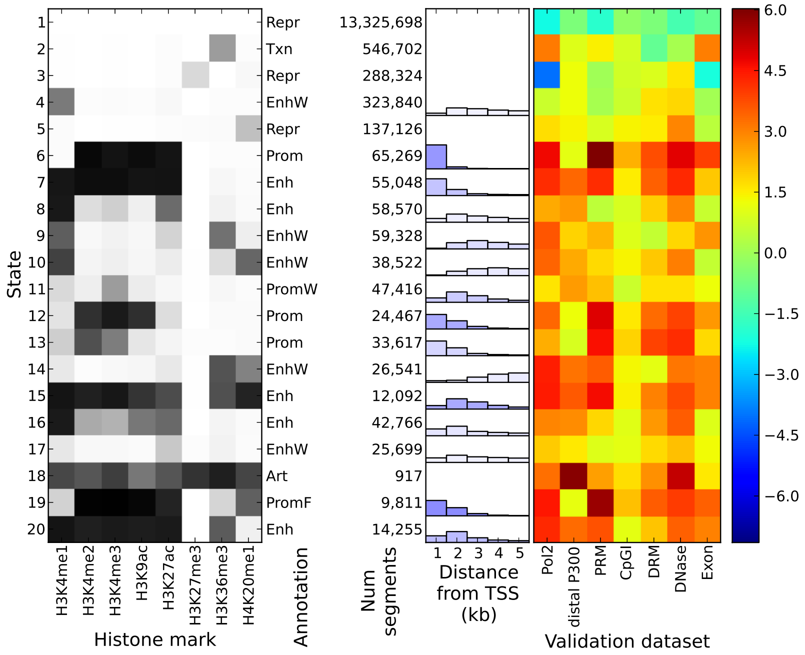

Figure S16 Spectacle emission matrix and state annotation for cell line K562 for 20 chromatin states. Refer to caption of Figure 1 in the main text.

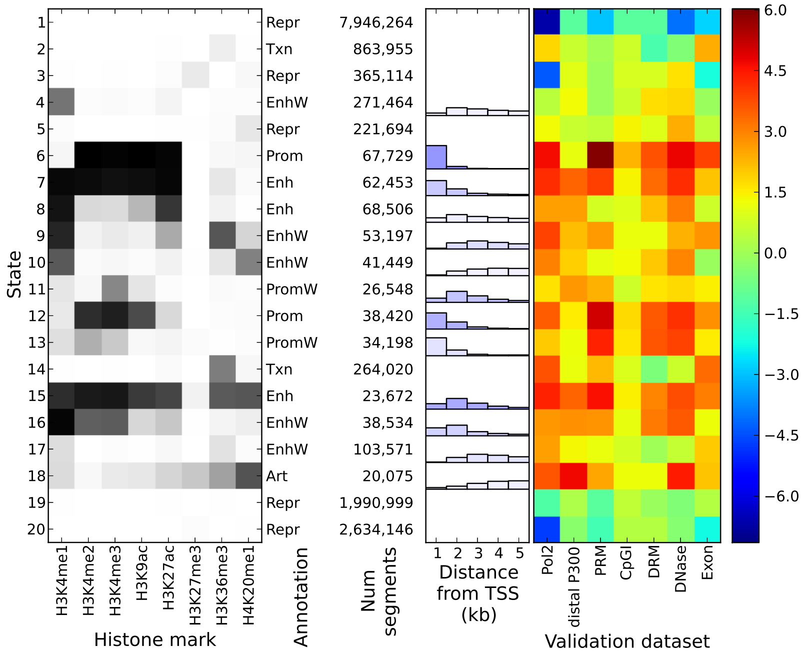

Figure S17 ChromHMM emission matrix and state annotation for cell line K562 for 20 chromatin states. Refer to caption of Figure 1 in the main text.

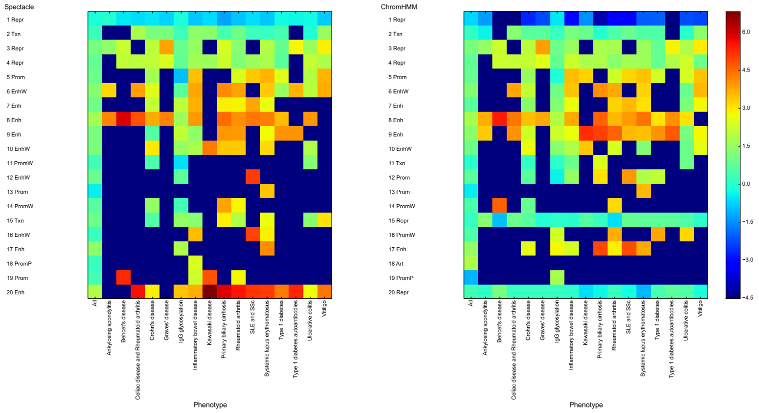

Figure S18 Chromatin states enriched with GWAS SNPs for all 16 autoimmune diseases that are most highly enriched in strong enhancer states predicted by Spectacle in GM12878. SLE and SSc stands for Systemic lupus erythematosus and Systemic sclerosis.

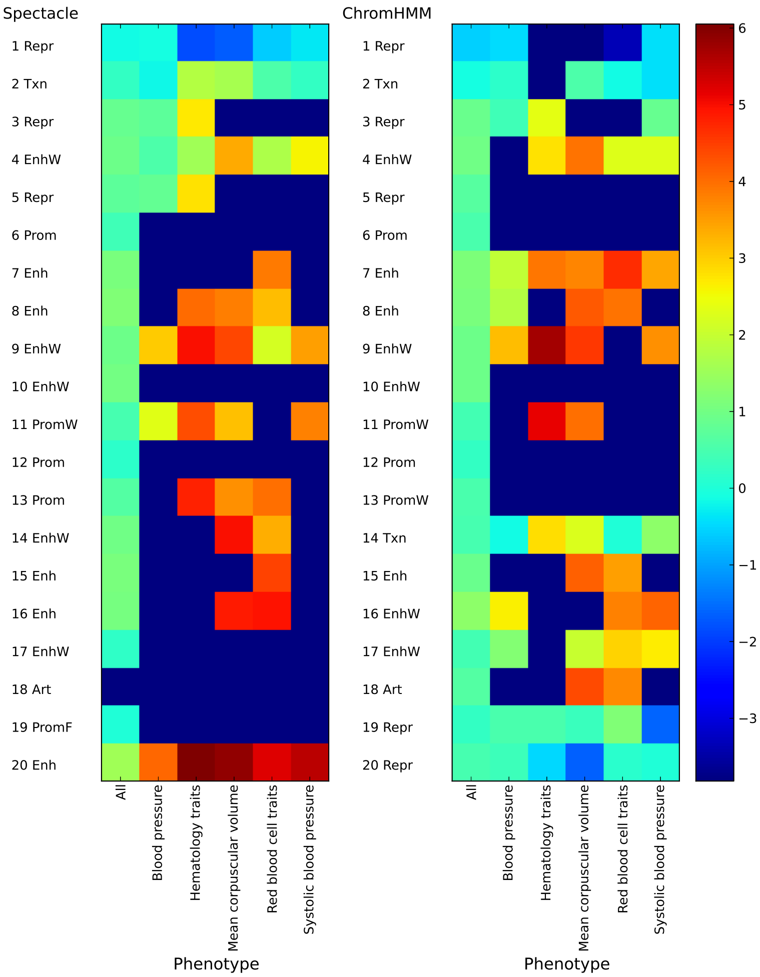

Figure S19 Chromatin states enriched with GWAS SNPs for five erythrocyte related phenotypes that are most highly enriched in strong enhancer states predicted by Spectacle in K562.

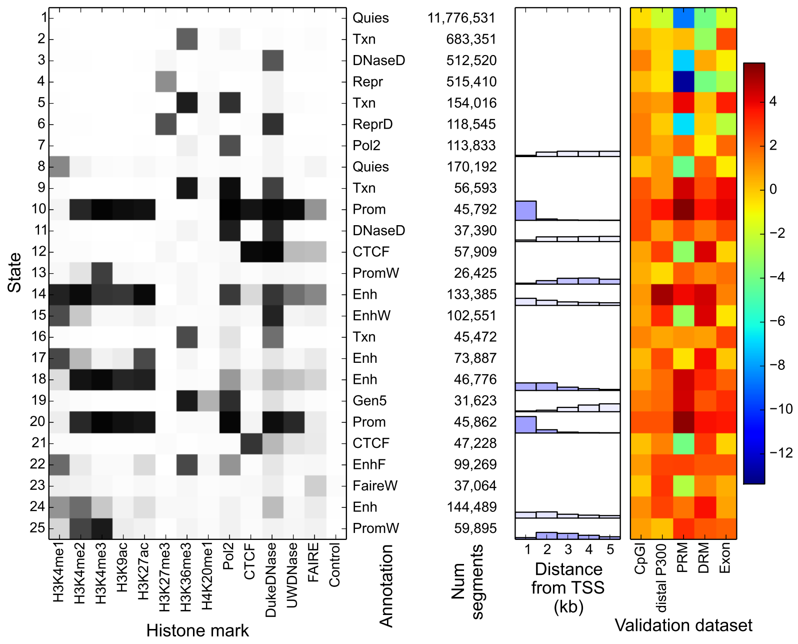

Figure S20 Spectacle emission matrix and state annotation for cell line GM12878 for 25 chromatin states. Refer to caption of Figure 1 in the main text.

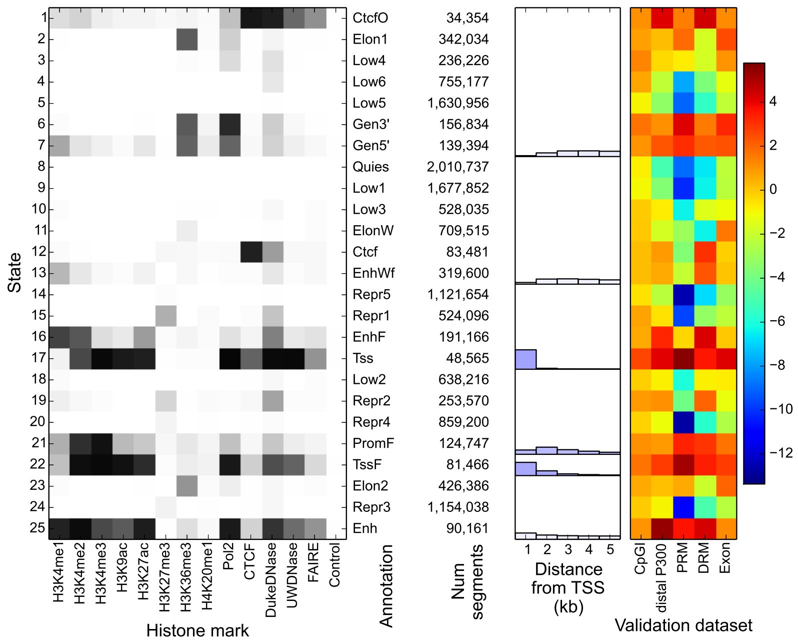

Figure S21 Segway emission matrix and state annotation for cell line GM12878 for 25 chromatin states. Refer to caption of Figure 1 in the main text.

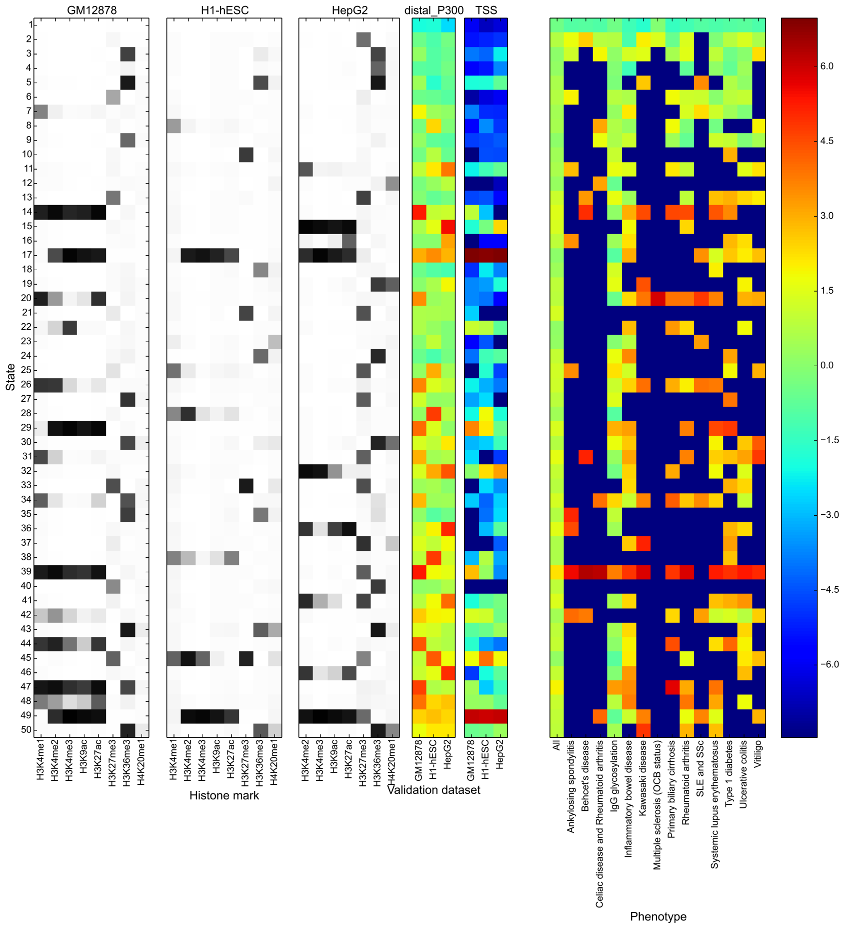

Figure S22 (Left) Spectacle emission matrix for combined epigenome datasets for three cell types (GM12878, H1-hESC and HepG2) for 50 chromatin states (values between 0 and 1 are colored from white to black). (Middle) enrichment of validated biological features for each cell type. (Right) chromatin states enriched with GWAS SNPs for autoimmune diseases. SLE and SSc stands for Systemic lupus erythematosus and Systemic sclerosis. For the middle and the right panels, enrichment and depletion are represented by the colors shown in the color bar.
